# Parsimonious Higher-Order Hidden Markov Models for Improved Array-CGH Analysis with Applications to *Arabidopsis thaliana*

**DOI:** 10.1371/journal.pcbi.1002286

**Published:** 2012-01-12

**Authors:** Michael Seifert, André Gohr, Marc Strickert, Ivo Grosse

**Affiliations:** 1 Department of Molecular Genetics, Leibniz Institute of Plant Genetics and Crop Plant Research (IPK), Gatersleben, Germany; 2 Institute of Computer Science, Martin Luther University Halle, Halle (Saale), Germany; 3 Science and Technology, University of Siegen, Siegen, Germany; University of Washington, United States of America

## Abstract

Array-based comparative genomic hybridization (Array-CGH) is an important technology in molecular biology for the detection of DNA copy number polymorphisms between closely related genomes. Hidden Markov Models (HMMs) are popular tools for the analysis of Array-CGH data, but current methods are only based on first-order HMMs having constrained abilities to model spatial dependencies between measurements of closely adjacent chromosomal regions. Here, we develop parsimonious higher-order HMMs enabling the interpolation between a mixture model ignoring spatial dependencies and a higher-order HMM exhaustively modeling spatial dependencies. We apply parsimonious higher-order HMMs to the analysis of Array-CGH data of the accessions C24 and Col-0 of the model plant *Arabidopsis thaliana*. We compare these models against first-order HMMs and other existing methods using a reference of known deletions and sequence deviations. We find that parsimonious higher-order HMMs clearly improve the identification of these polymorphisms. Moreover, we perform a functional analysis of identified polymorphisms revealing novel details of genomic differences between C24 and Col-0. Additional model evaluations are done on widely considered Array-CGH data of human cell lines indicating that parsimonious HMMs are also well-suited for the analysis of non-plant specific data. All these results indicate that parsimonious higher-order HMMs are useful for Array-CGH analyses. An implementation of parsimonious higher-order HMMs is available as part of the open source Java library Jstacs (www.jstacs.de/index.php/PHHMM).

## Author Summary

Array-based comparative genomics is a standard approach for the identification of DNA copy number polymorphisms between closely related genomes. The huge amounts of data produced by these experiments require efficient and accurate bioinformatics tools for the identification of copy number polymorphisms. Hidden Markov Models (HMMs) are frequently used for analyzing such data sets, but current models are based on first-order HMMs only having limited capabilities to model spatial dependencies between measurements of closely adjacent chromosomal regions. We develop parsimonious higher-order HMMs enabling the interpolation between a mixture model ignoring spatial dependencies and a higher-order HMM exhaustively modeling these dependencies to overcome this limitation. In an in-depth case study with *Arabidopsis thaliana*, we find that parsimonious higher-order HMMs clearly improve the identification of copy number polymorphisms in comparison to standard first-order HMMs and other frequently used methods. Functional analysis of identified polymorphisms revealed details of genomic differences between the accessions C24 and Col-0 of *Arabidopsis thaliana*. An additional study on human cell lines further indicates that parsimonious HMMs are well-suited for the analysis of Array-CGH data.

## Introduction

In recent years, the method of array-based comparative genomic hybridization (Array-CGH) [1]–[5] has been widely applied for the detection of DNA copy number polymorphisms between closely related genomes. Most Array-CGH studies have their focus in cancer research for the genome-wide identification of deletions and amplifications of genomic regions in tumor compared to healthy tissue [6]–[10]. With the availability of the genome sequence of the accession Columbia (Col-0) of the model plant *Arabidopsis thaliana* [11], studies comparing the genomes of different accessions have been performed using the Array-CGH approach to analyze evolutionary processes and phenotypic features at a molecular level [12]–[17]. All these studies require efficient bioinformatics methods for the precise identification of copy number polymorphisms from Array-CGH data.

Over the last years, a large number of different methods for the identification of copy number polymorphisms from Array-CGH data have been developed including approaches based on Gaussian mixture models [18], circular binary segmentation [19]–[21], genetic local search algorithms [22], [23], dynamic programming [24]–[26], hierarchical clustering [27], sparse Bayesian learning [28], variational methods [29], [30], smoothing techniques [31]–[34], regression models [35], [36], or wavelets [37], [38]. In-depth contributions to the comparison of different methods have been made by two studies [39], [40]. Selected well-performing methods have been made publicly available by webservers [41]–[44].

Despite these different methods, the identification of copy number polymorphisms by methods based on Hidden Markov Models (HMMs) is very popular [45]–[61] providing a natural way for modeling genomic spatial dependencies present in Array-CGH data. Most of these HMM -based methods use three up to six states with specific Gaussian emission densities for the modeling of Array-CGH measurements. Greater differences exist in learning principles used for adapting models to data. The Baum-Welch algorithm [62]–[65] has been used in [47], [48], [56], [59], [61] for estimating the parameters of the HMM by maximizing the likelihood without integrating prior knowledge on the distribution of Array-CGH measurements. Due to specific model extensions, numerical estimations of the likelihood have been considered in [50], [51]. Bayesian approaches using Markov Chain Monte Carlo simulations have been developed in [52]–[55], [58], a numerical Bayesian estimation has been applied in [57], and a Bayesian Baum-Welch algorithm has been utilized in [60]. All these Bayesian approaches enable the integration of prior knowledge on the distribution of Array-CGH measurements for improving the identification of copy number polymorphisms.

**Figure 1:**
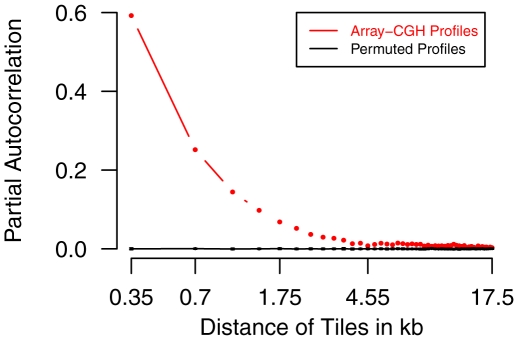
Spatial dependencies of measurements in Array-CGH profiles of *Arabidopsis thaliana*. The partial autocorrelation function characterizes spatial dependencies between measurements of adjacent chromosomal regions (tiles) in Array-CGH profiles. This function has been computed for the five chromosome-specific Array-CGH profiles by [103] comparing the genomes of the *Arabidopsis thaliana* accessions C24 and Col-0. The red curve represents the weighted mean partial autocorrelation function of the original Array-CGH profiles for increasing chromosomal distance of adjacent tiles in steps of 0.35 kb. The black curve represents mean values and standard deviations (both close to zero) of the mean weighted partial autocorrelation function for randomly permuted measurements in each of the five original Array-CGH profiles across 100 repeats. The significant presence of spatial dependencies of measurements in the Array-CGH profiles (red) compared to permuted profiles (black) motivates the modeling of such dependencies for the analysis of Array-CGH data.

A characteristic of all these HMMs is that they are based on the mathematical theory of standard first-order HMMs [65], [66]. This leads to a common limitation that all these HMMs can only model dependencies between Array-CGH measurements of two directly adjacent chromosomal regions. Yet, no attention has been paid to higher-order HMMs enabling the modeling of dependencies between a chromosomal region and its most recent predecessors that are clearly present in Array-CGH data (e.g. Figure 1).

In contrast to the broad usage of first-order HMMs in applied sciences [66]–[68], published applications of higher-order HMMs are relatively rare, but they have been demonstrated to be powerful extensions of first-order HMMs for several applications including speech recognition [69]–[76], image segmentation [77]–[79], robotic [80], handwriting recognition [81], or DNA and protein sequence analysis [82]–[85]. Extensions of the mathematical theory of first-order HMMs to higher-order HMMs are comprehensively described in [86]–[89]. The improved modeling of spatio-temporal dependencies by higher-order HMMs is realized by a more complex state-transition process defined on the basis of a higher-order Markov model reviewed in [90]. A limitation of this improved modeling is the exponential increase of transition parameters with increasing model order requiring growing amounts of data and computational resources for model training and evaluation. This has generally limited the usage of large model orders. Consequently, most existing studies have only focused on second-order HMMs [69]–[73], [78], [80], [82], [84].

To enable the usage of improved modeling characteristics of greater model orders by simultaneously overcoming the exponential increase of transition parameters, a fast incremental training has been developed in the domain of speech recognition [87], [91]. This heuristic algorithm iteratively increases the model order by only including transition parameters that are required for the representation of the training data. That has led to higher-order HMMs with reduced model complexities [87], [91], [92] and to mixed-order HMMs [93]–[95] reaching improved results in speech recognition in comparison to first-order HMMs and standard higher-order HMMs. In addition, a variable-length HMM has been developed to improve the modeling of motion capture data [96], [97]. The state-transition process of this model is defined by a variable memory Markov chain for which the transition parameters are determined by a minimum entropy criterion integrated into an extended Baum-Welch training. However, since implementations of both approaches for reducing the number of transition parameters are not publicly available and since algorithmic extensions would be necessary to enable the integration of prior knowledge, these models cannot directly be utilized for the analysis of Array-CGH data.

Here, we develop the novel model class of parsimonious higher-order HMMs enabling the interpolation between a mixture model ignoring spatial dependencies and a higher-order HMM exhaustively modeling spatial dependencies between measurements of closely adjacent chromosomal regions. This interpolation is realized by incorporating a dynamic programming approach [98], [99] into a specifically developed Bayesian Baum-Welch training algorithm enabling the integration of prior knowledge and a data-dependent reduction of transition parameters. Based on that interpolation, a parsimonious higher-order HMM can effectively model spatial dependencies between measurements of closely adjacent chromosomal regions.

In an in-depth case study with the model plant *Arabidopsis thaliana*, we apply parsimonious higher-order HMMs to compare the genomes of the accessions C24 and Col-0 based on a publicly available Array-CGH data set. This enables the identification of DNA polymorphisms (deletions or sequence deviations, amplifications) in C24 with respect to the reference genome of Col-0 [11]. We evaluate and compare parsimonious higher-order HMMs against standard first-order HMMs and other existing methods by making use of deletions or sequence deviations identified in an independent array-based resequencing experiment of C24 [100], [101]. Moreover, we perform a functional analysis of identified genomic differences revealing novel details of differences between C24 and Col-0, and we also consider widely used human cell lines [102] for additional model comparisons.

## Materials and Methods

In the materials part of this section, the Arabidopsis Array-CGH data set comparing the genomes of C24 and Col-0 is introduced and candidate regions of deletions or sequence deviations for model evaluation determined by an independent public resequencing experiment are considered. The model class of parsimonious higher-order HMMs is developed in the methods part of this section.

### Materials

In this section, the Arabidopsis Array-CGH data set is introduced and candidate regions of deletions or sequence deviations for model evaluation identified in resequencing data are considered.

#### Arabidopsis Array-CGH data

An Array-CGH data set by [103] (GEO accession: GSM611097) comparing the genomes of the accessions C24 and Col-0 of the model plant *A. thaliana* is used to investigate the identification of DNA polymorphisms (deletions or sequences deviations, amplifications) by different methods. This data set was measured on a NimbleGen tiling array representing the five chromosomes of the Col-0 reference genome [11] by 364,339 genomic regions (tiles). The length of each tile is about 60 bp. All tiles on the array are spaced nearly equidistantly along the chromosomes with a mean distance of about 350 bp between two adjacent tiles. Lengths of single-stranded DNA segments hybridized to this array were in the range of 300 bp up to 900 bp. The tiling array was processed using the NimbleScan software resulting in normalized measurements.

The measurement of tile 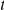 on chromosome 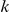 is given by the log-ratio 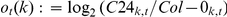 in dependency of the corresponding measured accession-specific fluorescent intensities 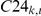 and 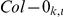. All log-ratios belonging to a chromosome 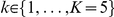 are summarized in an Array-CGH profile 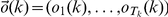 with 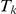 log-ratios represented in increasing order of the chromosomal locations of tiles.

Spatial dependencies between log-ratios on chromosomes are characterized in Figure 1. Tiles in close chromosomal proximity are highly correlated indicating that they have very similar measurements. These spatial dependencies between measurements of tiles in close chromosomal proximity (less than 5 kb) are most likely caused due to the lengths of single-stranded DNA fragments hybridized to the tiling array. Since the spacing between directly adjacent tiles on a chromosome is about 350 bp and because typically hybridized DNA fragments are having lengths up to 900 bp, it is expected that tiles in close chromosomal proximity are having very similar measurements.

**Figure 2:**
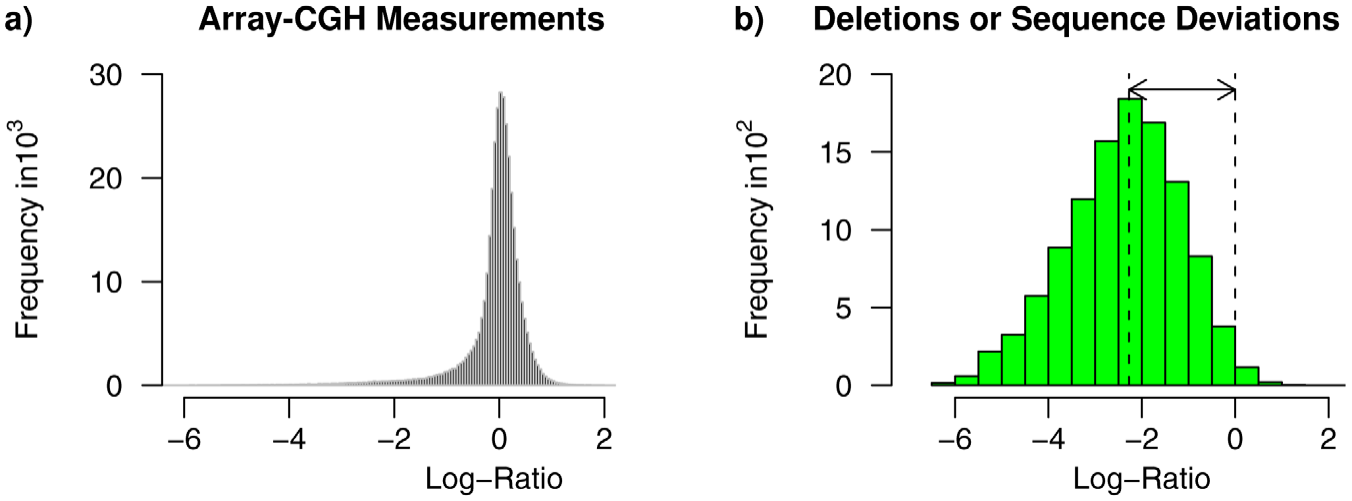
Characteristics of the *Arabidopsis thaliana* Array-CGH data set. **a**) Distribution of log-ratios measured for genomic regions in the Array-CGH data set by [103] comparing the genomes of the *Arabidopsis thaliana* accessions C24 and Col-0. The log-ratio of a genomic region characterizes changes in copy numbers (deletions or amplifications) or sequence deviations of this region in C24 in comparison to Col-0. Unchanged genomic regions between C24 and Col-0 have log-ratios close to zero. Deletions or sequence deviations of genomic regions in C24 have log-ratios much smaller than zero. Amplifications of genomic regions in C24 have log-ratios much greater than zero. **b**) Distribution of log-ratios of genomic regions (tiles) in the Array-CGH data set covered to at least 75% (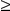
45 bp of 60 bp) by candidate regions of deletions or sequence deviations identified in [101] based on Affymetrix array-based resequencing data [100]. The large proportion of highly negative log-ratios indicates that these candidate regions are also present in the Array-CGH data set. Tiles covered by such candidate regions provide a useful resource for evaluating the identification of deletions or sequence deviations in the Array-CGH data set by different methods.

The distribution of log-ratios in the Array-CGH data set is shown in Figure 2a. Most of the tiles have log-ratios close to zero as expected for unchanged genomic regions between C24 and Col-0. A smaller proportion of tiles has log-ratios much smaller than zero as expected for deletions or sequence deviations for genomic regions in C24 compared to the corresponding regions in Col-0. Only a very small proportion of tiles has log-ratios much greater than zero as expected for amplifications of genomic regions in C24 in comparison to Col-0. The asymmetry of the log-ratio distribution is caused by the design of the tiling array exclusively representing genomic regions of the reference genome of Col-0 [11].

#### Arabidopsis resequencing data

An array-based Affymetrix resequencing experiment of C24 was performed in [100] for identifying single nucleotide polymorphisms and long stretches of deletions or sequence deviations. This experiment was further processed in [101] by the developed mPPR algorithm resulting in candidate regions of deletions or sequence deviations in C24 with respect to the reference genome sequence of Col-0 [11]. The identified candidate regions of deletions or sequence deviations have additionally been evaluated in [101] by comparisons against available sequence data and known deletions. This clearly indicated that these candidate regions are also present in other data sets. Thus, this data set provides a useful resource for the evaluation of deletions or sequence deviations identified by different models in the Array-CGH data set.

We used the determined candidate regions of deletions or sequence deviations from the resequencing experiment to identify each tile in the Array-CGH data set for which at least 75% of its nucleotides (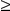
45 bp of 60 bp) are covered by candidate regions. This results in 11,025 tiles labeled as candidates for deletions or sequence deviations among the 364,339 tiles in the Array-CGH data set. As expected for potential deletions or sequence deviations in C24, most of these labeled tiles have log-ratios much less than zero in the Arabidopsis Array-CGH data set (Figure 2b). This indicates that deletions or sequence deviations determined in [101] are clearly present in the Arabidopsis Array-CGH data set and suggests that these candidate regions are useful for model evaluations.

### Methods

This section provides the basics of parsimonious higher-order HMMs. In the following, these models are introduced, a prior distribution for integrating prior knowledge into the training is specified, a model-specific Bayesian Baum-Welch training algorithm is developed, and details to the parameter initialization are given. Finally, a link to related work is given.

#### Parsimonious higher-order Hidden Markov Models

A parsimonious higher-order HMM with three states 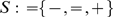 and Gaussian emissions is used for the analysis of Array-CGH profiles. Under consideration of the distribution of log-ratios in Array-CGH data (e.g. Figure 2a), the three states are defined to represent the following DNA polymorphisms. State ‘
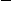
’ models deletions or sequence deviations with log-ratios much smaller than zero, state ‘
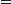
’ models unchanged regions with log-ratios close to zero, and amplifications with log-ratios much greater than zero are modeled by state ‘
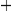
’. In contrast to other HMM -based methods like [48], [50], [52], [55], the states of the parsimonious higher-order HMM are not explicitly modeling specific genomic copy numbers, but the states are covering a broad range of state-specific log-ratios by making use of flexible Gaussian emission densities.

Each state 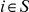 is characterized by a Gaussian emission density 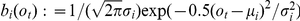 with state-specific mean 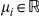 and standard deviation 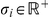 for modeling a log-ratio 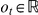. All emission parameters are summarized in the matrix 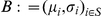.

The state underlying a chromosomal region 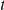 with corresponding log-ratio 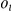 is denoted by 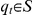. A state sequence 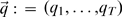 belonging to an Array-CGH profile 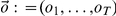 is assumed to be modeled by a parsimonious Markov model of order 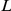 [98], [99]. This Markov model realizes the state-transition processes of the parsimonious higher-order HMM. The state-transition process is similar to that of a higher-order HMM [89] additionally enabling a data-dependent sharing of transition parameters for state-transitions from specific state-contexts.

In more detail, the state-transition process of a parsimonious HMM of order 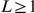 is defined by an initial state distribution 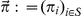 with initial state probability 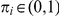 fulfilling 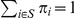 and a set of 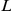 transition matrices 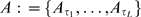. Each transition matrix 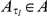 is defined on the basis of a state-context tree 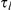 subdividing the product set of state-contexts 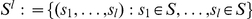 into disjoint sets of equivalent state-contexts. A specific set of equivalent state-contexts of 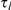 is denoted by 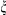. All state-contexts 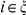 are assumed to share the identical transition probability 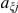 for a transition from each state-context in 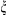 to a next state 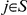. Thus, the parsimonious representation of state-contexts by sets of disjoint equivalent state-contexts reduces the total number of transition parameters of the model. Hence, the transition matrix 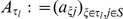 is defined by corresponding transition probabilities 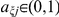 fulfilling 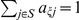.

Generally, the transition matrix 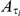 with 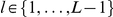 is used for the transition from the current state 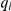 to the next state 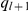 in dependency of the predecessor states 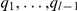, while the transition matrix 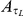 is used for the transition from 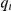 to 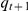 under consideration of the predecessor states 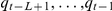 for all 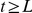.

**Figure 3:**
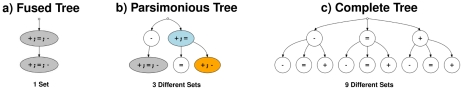
Examples of state-context trees. Selected state-context trees of height two representing different sets of disjoint sets of equivalent state-contexts of length two. The fused tree (**a)**) and the complete tree (**c)**) define marginal cases of state-context trees underlying a parsimonious higher-order HMM. Fused trees are underlying the mixture model, while complete trees are the basis of a higher-order HMM. The fused tree has the most parsimonious structure representing all state-contexts in one set of equivalent state-contexts, while the complete tree represents each state-context of length two by an individual set. The parsimonious tree (**b)**) with three disjoint sets of equivalent state-context has a complexity between the fused and the complete tree. More formally, each path from the root node at the top of a tree to a leaf node at the bottom of a tree represents a set of state-contexts defined to share common transition probabilities. The nodes directly under the root node of a tree represent possible current states, and the nodes under these nodes represent the corresponding predecessor states of the current state. Predecessor states have a specific influence on the state-transition from the current state to the next state depending on the type of the node. Exemplarily, some different types of nodes are highlighted in color. White nodes represent unfused nodes characterizing important states for a state-transition. Blue and orange nodes represent partially fused states of equal importance for a state-transition. Grey nodes represent completely fused nodes defining that the corresponding position in a state-context has no influence on a state-transition.

Exemplarily, three different types of state-context trees underlying a transition matrix 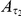 are illustrated in Figure 3. The completely fused tree (Figure 3a) assigns all state-contexts to one leaf node, the complete tree (Figure 3c) represents each state-context in a separate leaf node, and the parsimonious tree (Figure 3b) groups selected state-contexts together resulting in less leaf nodes than in a complete tree.

Completely fused trees are the basis for a mixture model of Gaussian densities (HMM of order zero) that does not model spatial dependencies between log-ratios in Array-CGH profiles. Complete trees are underlying a higher-order HMM exhaustively modeling spatial dependencies. Parsimonious trees provide the basis for a parsimonious higher-order HMM interpolating between a mixture model and a higher-order HMM. This interpolation poses the problem of selecting optimal state-context trees for an HMM. For a fixed set of states, the number of different state-context trees grows super-exponentially for increasing model order (Figure S1 in Text S1). Thus, each existing state-context tree cannot be analyzed separately. To overcome this, we compute optimal state-context trees by an efficient dynamic programming approach [98], [99] that has been incorporated into the Bayesian Baum-Welch training algorithm of the parsimonious higher-order HMM.

For identifying DNA polymorphisms in an Array-CGH profile, an extension of the standard state-posterior decoding algorithm [65] is used to compute the state-posterior probability 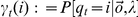 for quantifying the potential of a chromosomal region 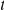 to be represented by a state 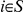. Details to the state-posterior decoding and the computation of state-posterior probabilities for a parsimonious HMM are given in [89]. The state-posterior probabilities are used to rank log-ratios according to their tendency of being modeled by a specific state of the model (e.g. state ‘
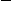
’ with respect to known deletions or sequence deviations from independent validation data). Additionally, these state-posterior probabilities can also be used to perform a decoding of individual measurements in an Array-CGH profile into the discrete states of the model by assigning the most likely state to each chromosomal region in an Array-CGH profile.

In summary, the parameters of the parsimonious higher-order HMM are denoted by 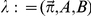 and the three-state architecture of this model is illustrated in Figure S2 in Text S1.

#### Prior distribution

A problem-specific characterization of the parameters of a parsimonious higher-order HMM 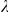 is achieved by integrating prior knowledge about Array-CGH profiles into the training. This is realized by specifying a prior distribution
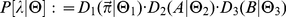
(1)for the parameters of the HMM 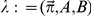 in dependency of the hyper-parameters 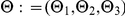. This prior is defined to be a product of independent priors for the initial state distribution 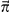, the set of transition matrices 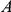, and the emission parameters 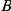. A conjugate prior distribution is chosen for each class of model parameters enabling the analytical parameter estimation during the training of a parsimonious higher-order HMM.

The prior distribution 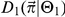 of the initial state distribution is defined to be a transformed Dirichlet distribution [104], and the prior distribution 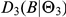 of the state-specific Gaussian emission densities is defined to be a product of Gaussian-Inverted-Gamma distributions [105]. These two prior distributions are the usual ones applied for HMMs (e.g. [66], [106]). Details to the prior of the initial state distribution and to the prior of the emission parameters are given in the section Prior distribution in Text S1.

In the following, the central transition prior 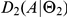 is specified in detail to provide the basics for computing the optimal state-context trees and corresponding transition parameters during the training. Since each transition matrix 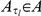 is defined by an underlying state-context tree 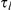 that represents different classes of equivalent state-contexts that share their transition parameters, the typically used Dirichlet prior for transition parameters of a fixed state-context must be re-defined to enable the evaluation of different structures of the underlying state-context tree. This is realized as follows.

The transition prior for the set of transition matrices 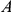 is defined by
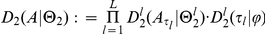
consisting of a product of transformed Dirichlet distributions 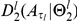 in combination with a tree structure prior 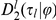 for each transition matrix 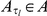. The corresponding hyper-parameters 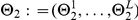 are specified with respect to each hyper-parameter matrix 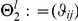 defining the pseudocounts 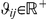 for a transition from a state-contexts 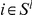 to a next state 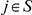.

The transformed Dirichlet distributions
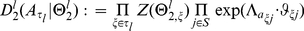
(2)define the prior for the transition parameters of the transition matrix 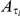 in dependency of the corresponding state-context tree 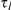. For each class of equivalent state-contexts 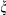 of the state-context tree 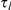 underlying the transition matrix 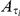, a transformed Dirichlet distribution is specified. Each transition probability 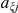 of 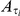 is parameterized in the log-space by 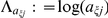. The corresponding hyper-parameter vector 
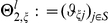
 with 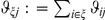 is defined with respect to 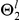, and the normalization constant is specified by 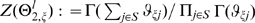 in dependency of the Gamma function 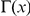 defined for all 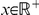.

The tree structure prior
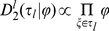
(3)is defined for rating the state-context tree 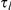 by its number of disjoint sets of equivalent state-contexts. During the training of a parsimonious higher-order HMM, the tree structure hyper-parameter 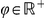 enables the regulation of the number of leaf nodes of a state-context tree influencing the tree structure of 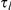. A fixed value of 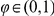 leads to a decreased value of the tree structure prior for an increasing number of leaf nodes, whereas a fixed value of 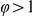 leads to a greater value of the tree structure prior for an increasing number of leaf nodes.

The choice of hyper-parameter values for the prior distribution of a parsimonious higher-order HMM should provide the basics for distinguishing between DNA polymorphisms and unchanged chromosomal regions in Array-CGH profiles. A histogram of log-ratios (e.g. Figure 2a) helps to characterize the states of the model. Different values of the hyper-parameter of the tree structure prior are chosen to enable the interpolation of the parsimonious higher-order HMM between a mixture model and a higher-order HMM. The interval of tree structure hyper-parameter values that has to be considered for this interpolation is depending on the size of the Array-CGH data set. Details to the chosen hyper-parameter values of the prior distribution are given in the section Prior distribution in Text S1.

#### Bayesian Baum-Welch training

A Bayesian Baum-Welch algorithm is developed to adapt the initial parameters of a parsimonious higher-order HMM to Array-CGH profiles. This algorithm extends the commonly used Baum-Welch algorithm [62]–[65] by integrating prior knowledge into the parameter estimation. The Bayesian Baum-Welch algorithm is an iterative training procedure belonging to the class of Expectation Maximization (EM) algorithms [107] for maximizing the log-posterior density of the parameters of a parsimonious higher-order HMM for a given data set. This is done by iteratively computing new parameters of the parsimonious higher-order HMM
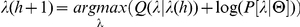
under consideration of its parameters 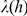 of the current iteration step 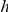 starting with initial parameters 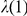. The parameter estimation is done based on Baum's auxiliary function 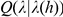 in combination with the logarithm of the prior distribution 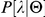 defined in (1).

Baum's auxiliary function is specified in [65] for a standard first-order HMM. Specific modifications are required for a parsimonious higher-order HMM due to the realization of the state-transition process by a parsimonious higher-order Markov model. In analogy to [65], Baum's auxiliary function is defined by
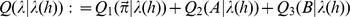
consisting of an auxiliary function for each class of model parameters. No modifications are required for the auxiliary function 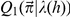 of the initial state distribution 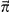 and for the auxiliary function 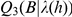 of the emission parameters 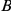. Details to these two functions and the corresponding parameter estimation are given in the section Bayesian Baum-Welch algorithm in Text S1.

The auxiliary function for the set of transition matrices 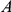 is given by
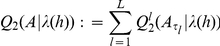
providing the basis for the computation of each state-context tree 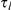 representing optimal disjoint sets of equivalent state-contexts of length 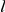 and corresponding transition probabilities of the transition matrix 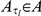. This requires the auxiliary function for each transition matrix 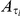 given by
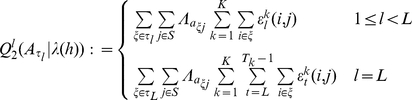
(4)under consideration of the log-transition probability 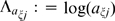 and the probability 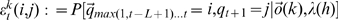 for a transition from state-context 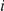 to next state 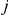 given the Array-CGH profile 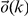 and the current parameters of the parsimonious higher-order HMM. The log-transition probability 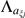 has to be estimated for the next parsimonious higher-order HMM 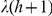. Each probability 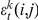 is computed under the parsimonious higher-order HMM 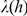 using extended versions of the standard Forward-Backward algorithm [65] as developed in [89]. Details for deriving 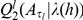 in (4) are provided in the section Bayesian Baum-Welch algorithm in Text S1.

For estimating the transition probabilities of transition matrix 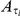, the logarithm of the transition prior 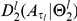 in (2) and the logarithm of the tree structure prior 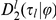 in (3) are added to the corresponding auxiliary function 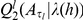 in (4). The resulting function is then maximized by a dynamic programming approach [98], [99] efficiently evaluating the set of all existing state-context trees 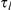. This results in an optimal state-context tree 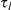 and a corresponding transition matrix 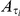 for the next parsimonious higher-order HMM 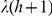. Details to the transition parameter estimation are given in the section Bayesian Baum-Welch algorithm in Text S1.

Generally, the applied dynamic programming approach starts with an initialization step having a computational complexity of 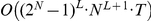 in dependency of the number of hidden states 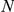 and the order 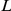 of the parsimonious HMM, and the length 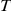 of a processed emission sequence. The term 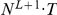 is standardly occurring for higher-order HMMs specifying the computational complexity required to compute all weights for the estimation of transition probabilities, and the term 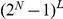 is specific for the dynamic programming approach used for the parsimonious higher-order HMMs.

The initialization step is followed by iteration steps that have a total computational complexity of 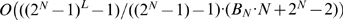. Here, 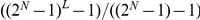 specifies the number of iteration steps, and the Bell number 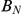 defines the number of partitions existing for 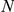 states growing faster than 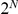 for 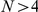. Details to the derivation of the computational complexities of the initialization and the iteration steps are given in the section Bayesian Baum-Welch algorithm in Text S1.

The estimation of new parameters 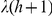 is iterated until the log-posterior density increases less than 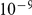 for two successive iteration steps of the Baysian Baum-Welch algorithm. This iterative scheme reaches at least a local optimum in dependency of the initial parameters 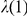 [107].

#### Model initialization

An initial parsimonious higher-order HMM has to distinguish between deletions or sequence deviations, unchanged chromosomal regions, and amplifications in an Array-CGH data set. A histogram of measured log-ratios (e.g. Figure 2a) assists to choose initial parameters for the state-specific Gaussian emission densities characterizing the three states of the model in Figure S2 in Text S1.

For the Array-CGH data set comparing the genomes of C24 and Col-0, the initial means of the state-specific Gaussian emission densities are set to 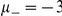, 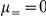, and 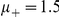. The initial standard deviation of the Gaussian emission density of each state 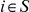 is set to 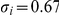 according to the standard deviation of log-ratios in the Array-CGH data set.

The initial state distribution 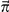 is sampled from the prior distribution of the initial state distribution. Each initial transition matrix 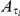 is sampled from its corresponding transition prior distribution by assuming an underlying complete state-context tree (e.g. Figure 3c) of a higher-order HMM. That means, a parsimonious higher-order HMM is initially representing a corresponding higher-order HMM.

Parsimonious HMMs of order one up to five have been considered for the analysis of the Array-CGH data set. For each model order, forty different model complexities ranging from the mixture model up to the corresponding higher-order HMM have been evaluated by using forty different values of the hyper-parameter 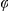 of the tree structure prior in Equation (3). Details to the chosen hyper-parameter values are given in the section Prior distribution in Text S1. For each of these forty different hyper-parameter values, twenty different initial models have been adapted to the Array-CGH data set using the Bayesian Baum-Welch training. Thus, in total 800 different models were computed for each model order. The best performing models with clearly reduced model complexities in comparison to higher-order HMMs were obtained for 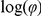 in the range of −100 to 0.

Generally, apart from this in-depth study considering the Arabidopsis Array-CGH data set, a parsimonious higher-order HMM can be specified for the analysis of Array-CGH data by choosing appropriate values for the mean values of the Gaussian emission densities of the states ‘
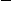
’ and ‘
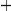
’. The mean value of the Gaussian emission density of state ‘
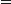
’ can be assumed to be zero, because unchanged chromosomal regions are expected to have log-ratios of about zero. The standard deviations of the state-specific Gaussian emission densities can be initially set to the standard deviations of the considered Array-CGH data set. Using the pre-defined hyper-parameter values for the prior distributions (see section Prior distribution in Text S1), good-performing models have been obtained on Arabidopsis and human Array-CGH profiles. Especially for model orders greater than one, good-performing models with a clearly reduced model complexity in comparison to the corresponding higher-order HMM have been obtained for choosing the tree structure hyper-parameter value 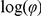 in the range of −100 to 0. This initialization concept is realized in the provided software and further specific hints are given in the corresponding documentation.

#### Related work in other domains: Variable-length Hidden Markov Models

Related to parsimonious higher-order HMMs, a variable-length HMM was developed in [96], [97] for the analysis of motion capture data of modern human dance. The state-transition process of the variable-length HMM is defined by a variable memory Markov chain. The transition parameters of this Markov chain are determined by a minimum entropy criterion based on the Kullback-Leibler divergence integrated into an extended Baum-Welch training. The minimum entropy criterion is used for pruning or growing the state-contexts that are underlying the state-transition process. The Baum-Welch algorithm developed for the variable-length HMM does not enable the integration of prior knowledge into the training of model parameters.

In contrast to this, the parsimonious higher-order HMM is trained by a Bayesian Baum-Welch algorithm enabling the integration of prior knowledge. Especially for HMM -based analysis of DNA microarray data, the modeling of prior knowledge can have a substantial impact on the quality of analysis results [106]. Generally, the concept of pruning or growing of state-contexts developed for the variable-length HMM is related to the concept of determining sets of equivalent state-contexts forming the basis of the parsimonious higher-order HMM. The state-transition process of the parsimonious higher-order HMM is more flexible enabling shared transition probabilities due to fusions of nodes in the underlying state-context tree. This allows to model dependencies between non-directly adjacent states for which the intermediate states are not or only partially contributing to these dependencies. That is exemplarily illustrated in Figure 3b in which the right tree branch contains a partially fused node with non-completely fused child nodes. Such dependencies cannot be modeled by a variable-length HMM because pruning or growing only enables to shorten or extend state-contexts but not to fuse states.

## Results/Discussion

In this section, first the modeling of spatial dependencies between Arabidopsis Array-CGH measurements is investigated to choose a range of model orders for parsimonious HMMs. Based on this, parsimonious HMMs of different model complexity are compared regarding their ability to identify deletions or sequence deviations in the Arabidopsis Array-CGH data set. Additionally, parsimonious HMMs are compared to existing methods utilizing the Arabidopsis and human cell lines Array-CGH data. Finally, a detailed functional classification of identified copy number polymorphisms or sequence deviations is made to investigate potential functions of genomic regions in which the genomes of C24 and Col-0 differ.

### Choice of Model Order

The modeling of the partial autocorrelation function [108] of the Arabidopsis Array-CGH profiles by higher-order HMMs was initially studied to determine a range of model orders for an in-depth analysis by parsimonious HMMs. The partial autocorrelation function quantifies linear dependencies between measurements of chromosomal regions in close chromosomal proximity for an increasing distance of regions. As shown in Figure 1, such dependencies are clearly present in the Arabidopsis Array-CGH profiles motivating the application of HMMs of different model orders for modeling of these dependencies.

Initially, HMMs of order zero up to five were trained on the Arabidopsis Array-CGH profiles using the Bayesian Baum-Welch algorithm. Next, each HMM was used to sample 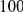 artificial profiles with 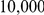 log-ratios. These profiles were used to compute the mean partial autocorrelation function modeled by each HMM.

As expected from theory, the HMM of order zero (mixture model) does not model dependencies between log-ratios in any chromosomal distance. The first-order HMM shows a clear improvement in comparison to the mixture model, but especially HMMs of order three up to five reached the best, nearly identical approximation of the partial autocorrelation function of Array-CGH profiles. A better modeling of the partial autocorrelation function by higher-order HMMs is expected from theory because of their more complex state-transition processes enabling an improved modeling of spatial dependencies compared to HMMs with a smaller model order. Still, none of these HMMs was able to perfectly approximate the partial autocorrelation structure of the Array-CGH profiles. But, despite of that, this study helped to determine a range of model orders for further analyses. The results of this study are summarized in Figure S3 in Text S1.

Based on this initial study with higher-order HMMs, parsimonious HMMs of order one up to five are subsequently investigated in detailed studies to analyze their abilities to identify DNA polymorphisms between C24 and Col-0.

### Stringent Identification of Deletions or Sequence Deviations

An Array-CGH data set by [103] comparing the genomes of the accessions C24 and Col-0 of *A. thaliana* is used to identify polymorphic regions between both genomes by parsimonious higher-order HMMs. These models are evaluated based on deletions or sequence deviations determined in [101] for the genome of C24 in comparison to the reference genome of Col-0 using publicly available array-based resequencing data [100]. The mapping of these polymorphic regions to corresponding chromosomal regions in the Array-CGH data set shows an obvious coupling with potential deletions or sequence deviations present in the Array-CGH data set (Figure 2b). These potential deletions or sequence deviations are used as reference for model comparisons.

**Figure 4:**
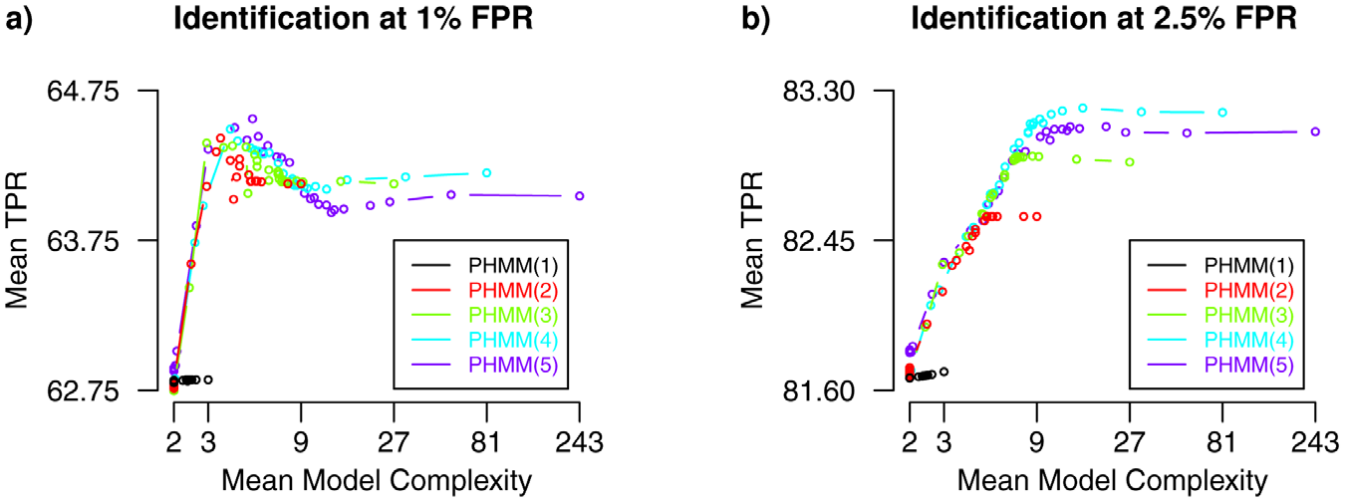
Identification of deletions and sequence deviations in the Arabidopsis Array-CGH data set by parsimonious HMMs. Curves of mean true-positive-rates (TPRs) for the identification of candidate regions of deletions or sequence deviations at a fixed false-positive-rate (FPR) of 1% (**a)**) and of 2.5% (**b)**) obtained by parsimonious HMMs of order 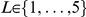 of different model complexities across twenty different initializations. The rightmost point of each curve of parsimonious HMMs of order 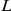 (PHMM(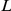)) represents the corresponding higher-order HMM of order 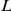 with highest model complexity of 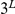 leaf nodes in the state-context tree underlying the transition matrix 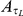. The rightmost point of the black curve represents the standard first-order HMM. Standard deviations of the mean TPRs are shown in Figure S4 in Text S1. At both levels of FPRs, parsimonious higher-order HMMs are clearly better than parsimonious HMMs of order one including the standard first-order HMM. At the level of 1% FPR, parsimonious higher-order HMMs with a mean model complexity in the range of 3 up to 9 also identify deletions or sequence deviations better than higher-order HMMs. At 2.5% FPR, clearly reduced model complexities are sufficient to reach identifications of deletions or sequence deviations by parsimonious higher-order HMMs comparable or slightly better than corresponding higher-order HMMs.

Parsimonious higher-order HMMs of different model complexities were adapted to the Array-CGH data using the developed Bayesian Baum-Welch training. For each model, all chromosomal regions in the Array-CGH data set were ranked in decreasing order of their state-posterior probabilities of state ‘
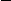
’ modeling deletions or sequence deviations. Using the knowledge about potential deletions or sequence deviations in the Array-CGH data set, the identification of these polymorphic regions was quantified for each model in terms of the true-positive-rate (TPR) at 1% false-positive-rate (FPR). The mean TPRs obtained for twenty different initializations of each model at 1% FPR are shown in Figure 4a (see Figure S4a in Text S1 for standard deviations of TPRs and see Figure S5a in Text S1 for FPRs at fixed TPR). The application of parsimonious higher-order HMMs has clearly improved the identification of deletions or sequence deviations in comparison to the standard first-order HMM. Moreover, parsimonious higher-order HMMs with much smaller model complexities than corresponding higher-order HMMs can also reach a clearly improved accuracy for identifying polymorphic regions in comparison to corresponding higher-order models. The best parsimonious higher-order HMMs have model complexities in the range of 3 up to 9 leaves. This range of model complexities includes parsimonious HMMs of order two up to five that nearly reach the same performance for identifying deletions or sequence deviations. State-context trees underlying well-performing parsimonious HMMs of order three up to five are clearly reduced leading to model complexities comparable with that of parsimonious second-order HMMs. Thus, not all higher-order dependencies are required for reaching a good performance at the stringent level of 1% FPR.

Similar results are shown in Figure S6a in Text S1 using a less restrictive mapping of the independently determined deletions or sequence deviations from [101] to the Array-CGH data set for model comparisons.

### Less Stringent Identification of Deletions or Sequence Deviations

Parsimonious higher-order HMMs have initially been compared against the standard first-order HMM and higher-order HMMs at a stringent FPR of 1%. Next, these models are compared at a less stringent FPR of 2.5%. That leads to an identification of deletions or sequence deviations comparable with those obtained by applying the state-posterior decoding algorithm [65], [89] that computes for each chromosomal region in the Array-CGH data set the most likely state under the given model. The results are shown in Figure 4b (see Figure S4b in Text S1 for standard deviations of TPRs and see Figure S5b in Text S1 for FPRs at fixed TPR).

Generally, parsimonious higher-order HMMs reach a higher accuracy for the identification of deletions or sequence deviations than the standard first-order HMM. The best parsimonious higher-order HMMs also reach an accuracy that is comparable or slightly better than that of corresponding higher-order HMMs. This accuracy is obtained at much lower model complexities than for higher-order HMMs. That can become particularly useful for avoiding overfitting in small data.

In comparison to the results at 1% FPR, the complexity of the best models is more shifted into the range of 9 to 27 leaves at 2.5% FPR (Figure 4 and Figure S4 in Text S1). This indicates that the identification of polymorphic regions is more complicated. Because at a higher FPR, the Array-CGH measurements of additionally identified polymorphic regions are more similar to that of non-polymorphic regions. These difficulties tend to be managed best by parsimonious higher-order HMMs. The best models in Figure 4b are among the fourth-order parsimonious higher-order HMMs.

**Figure 5:**
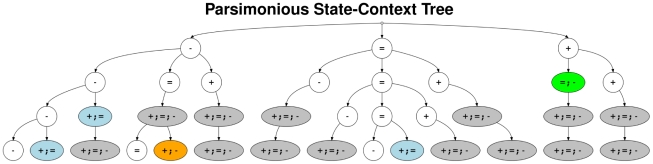
State-context tree of a parsimonious fourth-order HMM. Parsimonious state-context tree selected among the best parsimonious HMMs of order four at a fixed FPR of 2.5% in Figure 4b. Each path from the root node at the top of the tree to a leaf node at the bottom of the tree represents a set of state-contexts defined to share common transition parameters in the transition matrix 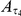 of the selected model. The three nodes directly under the root node represent the possible current states of the selected parsimonious fourth-order HMM, and the subtrees under these three nodes represent the influence of predecessor states on a state-transition from one of these current states to a next state. Fusions of nodes are highlighted in different colors. White nodes represent unfused nodes characterizing important states for a state-transition. Blue, orange, and green nodes represent partially fused states of equal importance for a state-transition. Grey nodes represent completely fused nodes defining that the corresponding position in a state-context has no influence on a state-transition. The states ‘
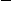
’ and ‘
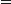
’ of the selected model are still representing some fourth-order transition probabilities, whereas only second-order transition probabilities remain for state ‘
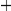
’. The selected parsimonious fourth-order HMM has a model complexity of 14 leaf nodes leading to 42 different transition parameters in 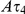. This is much less than for a corresponding fourth-order HMM with 81 leaf nodes in a complete state-context tree representing 243 transition parameters.

A tree structure of one of the best models is shown in Figure 5. The underlying parsimonious fourth-order HMM has still some specific fourth-order transition probabilities for the states ‘
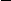
’ (deletion or sequence deviation) and ‘
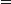
’ (non-polymorphic), whereas those of state ‘
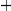
’ (amplification) are completely reduced to second-order transition probabilities. This unbalanced reduction of transition parameters tends to be coupled with the asymmetry of the Array-CGH measurement distribution in Figure 2a. Most of the chromosomal regions in the Array-CGH data set are non-polymorphic, a small proportion tends to be deleted or affected by sequence deviations, whereas only a very small proportion of regions tends to be amplified. The tree structure indicates that these tendencies are transferred to the number of transition parameters per state. This parsimonious fourth-order HMM is considered in all further studies with the Arabidopsis Array-CGH data set because of its good performance at the level of 2.5% FPR comparable with the results obtained by applying the state-posterior decoding algorithm enabling an in-depth analyses of genomic differences between C24 and Col-0.

Generally, similar tendencies like shown in Figure 4b are also present in Figure S6b in Text S1 considering a less restrictive mapping of the independently determined deletions or sequence deviations from [101] to the Arabidopsis Array-CGH data set for model comparisons.

### Comparison to Existing Methods

Here, the well-performing parsimonious fourth-order HMM is compared against other existing methods on the Arabidopsis data set. Then, another widely considered human cell lines data set by [102] is used for additional model comparisons. Subsequent to this, the focus is on comparative genomics of the accessions C24 and Col-0 of *A. thaliana*.

#### Comparison on Arabidopsis data

Next, the parsimonious fourth-order HMM with underlying tree structure shown in Figure 5 is compared to other existing methods for analyzing Array-CGH data. The standard method for the analysis of the Array-CGH data set measured on a NimbleGen tiling array is the segMNT algorithm [21]. Additionally, all eight methods provided by the ADaCGH webserver [43] including the best performing methods of two in-depth comparison studies [39], [40] were applied to the Arabidopsis Array-CGH data set. From these eight methods, only ACE [33], CBS [20], FHMM [48], and GLAD [32] were able to manage the huge number of Array-CGH measurements. Besides FHMM, also three other methods based on first-order HMMs were considered for the comparison including wuHMM [56] and two Bayesian methods RJaCGH [55] and GHMM [52]. All methods were applied to the Arabidopsis Array-CGH data set using standard settings.

The identification of deletions or sequence deviations by these methods is compared against the predictions of the parsimonious fourth-order HMM with respect to the known potential deletions or sequence deviations characterized in Figure 2b. For this comparison, a receiver operating characteristic (ROC) curve was computed for each method. This was done by ranking all chromosomal regions of the Array-CGH data set according to their method-specific scores enabling the evaluation of identified deletions or sequence deviations under consideration of known potential polymorphic regions.

**Figure 6:**
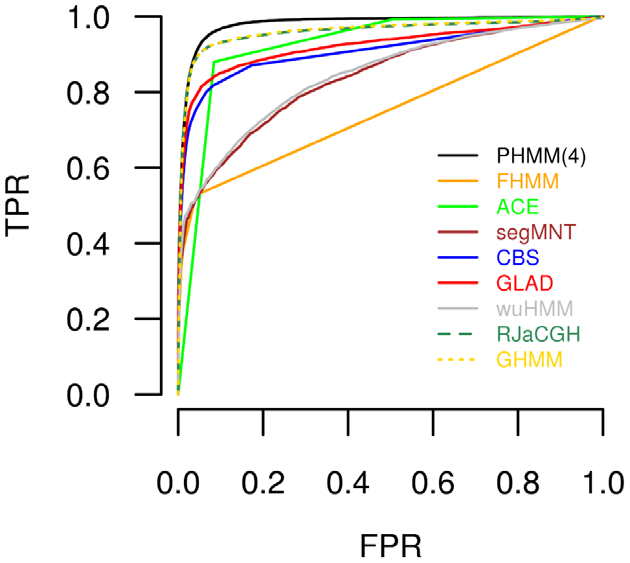
Comparison of a parsimonious fourth-order HMM to existing methods on the Arabidopsis Array-CGH data set. Receiver operating characteristic (ROC) curves for comparing the identification of deletions or sequence deviations in the Array-CGH data set. ROC curves are shown for FHMM, ACE, CBS, and GLAD of the ADaCGH webserver [43], segMNT [21], wuHMM [56], GHMM [52], RJaCGH [55] and the parsimonious fourth-order HMM with underlying state-context tree in Figure 5. The parsimonious fourth-order HMM reaches the best identification of deletions and sequence deviations (black).

The ROC curves are shown in Figure 6. The two Bayesian HMMs RJaCGH and GHMM identify deletions or sequence deviations with a nearly identical accuracy and better than wuHMM and all methods provided by the ADaCGH webserver. This is further improved by the parsimonious fourth-order HMM identifying chromosomal regions affected by deletions or sequence deviations with higher accuracy than all other methods. Comparable results were obtained considering a less restrictive mapping of identified deletions or sequence deviations from [101] to the Array-CGH data set (Figure S7 in Text S1). The improved performance of the parsimonious fourth-order HMM for identifying deletions or sequence deviations in comparison to the standard first-order HMM is highlighted in the direct comparison shown in Figure S8 in Text S1. Again all these findings indicate that parsimonious higher-order HMMs are useful for the analysis of Array-CGH data.

Comparing the different HMM-based methods by the number of hidden states required for modeling chromosomal aberrations in the Arabidopsis data set, wuHMM and FHMM both determined seven states, RJaCGH used six, and GHMM and the parsimonious HMM required only three states for reaching the reported performance. Supported by the best identification of deletions or sequence deviations, this indicates that the three states of the parsimonious HMM are flexible enough for modeling complex Arabidopsis Array-CGH profiles.

The two good-performing Bayesian HMMs RJaCGH and GHMM had substantially different run-times. RJaCGH required 30 hours and 42 minutes for analyzing the Arabidopsis data set, while GHMM only required about 24 minutes. An overview of run-times of all methods is given in Table 1. The training of a parsimonious first-order HMM on the Arabidopsis data set took about 2 minutes. This time is increased by a factor of three (number of hidden states) for increasing model order leading to a training time of about 54 minutes for the parsimonious fourth-order HMM. Using such a trained parsimonious HMM, analyses of data sets with a similar measurement distribution (e.g. comparisons of other accessions against Col-0) can be obtained in less than five minutes.

**Table 1:** Method run-times on the Arabidopsis Array-CGH data set.

Shortcut	Method	Reference	Computing time
wuHMM	First-order HMM	[56]	8 min
GHMM	Bayesian first-order HMM	[52]	24 min
PHMM	Parsimonious fourth-order HMM	see Methods	54 min
CBS	Circular Binary Segmentation	[20]	1 h 18 min
ACE	Analysis of Copy Errors	[33]	4 h 14 min
GLAD	Gain and Loss Analysis of DNA	[32]	4 h 19 min
FHMM	First-order HMM	[48]	5 h 04 min
RJaCGH	Bayesian first-order HMM	[55]	30 h 42 min

Run-times in hours/minutes required for the analysis of the Arabidopsis Array-CGH data set by the different methods. All methods except GHMM, PHMM, wuHMM, and RJaCGH were run on the ADaCGH web-server [43] (AMD Opteron 2.2 GHz CPU with 6 GB RAM). The other methods GHMM, PHMM, wuHMM, and RJaCGH were run on a standard desktop computer with Intel CPU T9500 2.6 GHz and 4 GB RAM.

In summary, this study further illustrated that parsimonious higher-order HMMs can outperform existing methods and are well-suited for analyzing Arabidopsis Array-CGH data. It should also be noted that experts of specific methods might be able to improve the results of individual methods by fine-tuning of specific parameters. Still, parsimonious higher-order HMMs represent an important contribution to the field of Array-CGH data analysis because they combine improved modeling of spatial dependencies with the integration of prior knowledge and because these models have reached a good performance on the Arabidopsis Array-CGH data.

#### Comparison on human cell lines

Additional model evaluations were also done on Array-CGH data of human cell lines [102] frequently considered in other model comparison studies like e.g. [20], [32], [48], [52], [55]. Details to the cell lines and the study are given in the section Model evaluations on human cell lines in Text S1. Using standard settings, six methods from the ADaCGH webserver [43], wuHMM [56], RJaCGH [55], and GHMM [52] were compared against a parsimonious first-order HMM to evaluate the identification of known trisomies and monosomies in the human cell lines. The resulting ROC curves are shown in Figure S9 in Text S1. The parsimonious first-order HMM, but also both Bayesian HMMs RJaCGH and GHMM reach the best, nearly perfect identification of known chromosomal aberrations in the individual human cell lines.

Considering the run-times on the human data set with about 17.5 times less measurements than in the Arabidopsis data set, RJaCGH required the longest time with about seventy minutes. Both, the GHMM and the parsimonious first-order HMM required only about one minute for analyzing the human cell lines. A summary of run-times of the ten different tested methods is given in Table S1 in Text S1. This additional study indicates that parsimonious HMMs are also useful for the analysis of non-plant-specific Array-CGH data.

### Functional Analysis of Genomic Differences between C24 and Col-0

The genome annotation of the reference genome of Col-0 provides the opportunity to investigate what is functionally behind chromosomal regions where the genomes of C24 and Col-0 differ. The parsimonious fourth-order HMM with underlying parsimonious tree structure in Figure 5 was applied to identify polymorphic regions in the Arabidopsis Array-CGH data set. The state-posterior decoding algorithm [65], [89] was used to classify each chromosomal region in the Array-CGH data set either as a deletion or sequence deviation, as unchanged, or as an amplification in C24 with respect to the reference genome of Col-0. This algorithm assigns the most likely state of the three-state architecture of the HMM (Figure S2 in Text S1) to each chromosomal region measured in the Array-CGH data set. The identification of deletions or sequence deviations by state-posterior decoding is comparable to that shown in Figure 4b.

In total, about 4.7% (17,306 of 364,339) of all chromosomal regions of the reference genome of Col-0 were identified as being affected by deletions or sequence deviations in the genome of C24, and about 0.2% (855 of 364,339) of all chromosomal regions were identified as amplified in C24 (Table S1). This asymmetry in predictions is expected from the distribution of measurements in the Array-CGH data set (Figure 2) reflecting the design of the tiling array that only represents chromosomal regions present in the reference genome of Col-0 [11]. Of the 17,306 chromosomal regions identified as being affected by deletions or sequence deviations, 2,647 are singletons consisting of only one tile and 76.5% of these singletons are containing a micro-deletion or sequence deviation in C24 compared to Col-0 that is covering at least 40% of the underlying tile. In all, genomic regions affected by deletions or sequence deviations represent about 5.59 Mb of the Col-0 reference genome. This is in good accordance with the findings in [100], [101]. Subsequently, all identified genomic differences are analyzed in detail.

#### Genome annotation analysis

**Figure 7:**
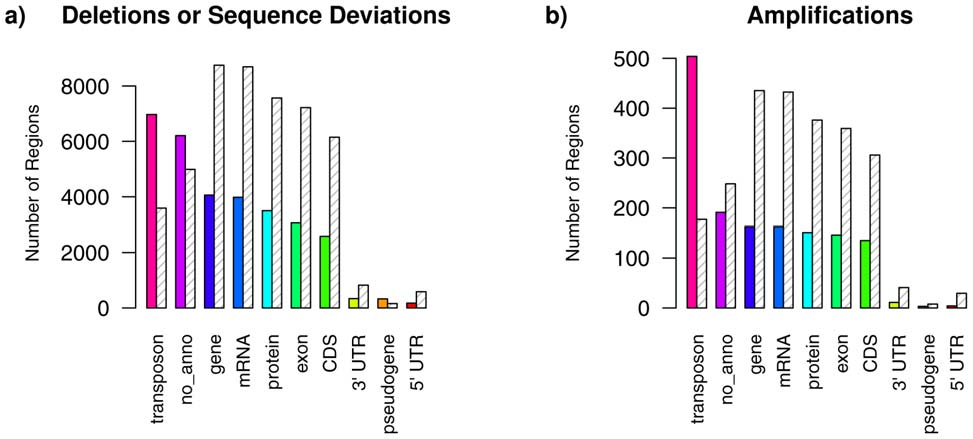
Functional classification of genomic differences in the Arabidopsis Array-CGH data set. Functional classification of the 17,306 tiles identified to be affected by deletions or sequence deviations (**a)**) and of the 855 tiles identified to be affected by amplifications (**b)**) in C24 in comparison to Col-0 according to the categories of the TAIR8 genome annotation. Colored bars show the counts in each category obtained for the Array-CGH data set by using the state-posterior decodings of the parsimonious fourth-order HMM with underlying state-context tree structure in Figure 5. Grey dashed bars represent the mean values of counts in each category obtained by sampling 500 times 17,306 tiles (or 855 tiles) from the total number of tiles in the Array-CGH data set. All counts in the different categories obtained for the Array-CGH data set, except ‘pseudogene’ for tiles identified as amplified, differ significantly from the random counts with p-values less than 0.01.

Chromosomal regions identified as being affected by deletions or sequence deviations and regions identified as being affected by amplifications were analyzed separately using the *Arabidopsis* Information Resource (TAIR8) genome annotation of Col-0 [109]. The results of these functional categorizations are summarized in Figure 7.

By definition, the TAIR8 categories are not completely disjoint meaning that each chromosomal region can have annotations in more than one category (e.g. chromosomal regions within genes). Comparisons of the identified polymorphic regions in C24 to randomly chosen control sets revealed that a significant proportion of chromosomal regions affected by deletions or sequence deviations and also that regions affected by amplifications are caused by transposons. Such mobile genomic elements were also identified to be involved in rearrangements of the genomes of other accessions of *A. thaliana* [17], [100], [110]. Moreover, genic regions as well as 5′ and 3′ untranslated regions (UTRs) are significantly less affected by amplifications and deletions or sequence variations.

Thus, genomic differences between C24 and Col-0 do not occur randomly because transposons differ more than other parts of the genome. These results are also supported by the finding that transposons change faster than genes [111].

#### Ontology classification of genes

Ontology classification was performed for genes affected by amplifications and for genes affected by deletions or sequence deviations using the MIPS Functional Catalogue [112] to investigate if specific functional categories of genes are over-represented.

No prevalence of any functional category was found for the 39 genes affected by amplifications. In contrast to this, among the 1,675 genes affected by deletions or sequence deviations, five significantly over-represented functional clusters of genes with p-values less than 5

10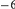 were identified. The first cluster comprises 104 genes with functions in ATP-binding, the second cluster contains 109 genes with functions in cellular communication and signal transduction, the third cluster represents 127 genes playing a role in cell rescue, defense and virulence, the fourth cluster contains 5 genes encoding for N-actetylglucosamine deacetylases, and the fifth cluster comprises 541 unclassified proteins.

In coincidence with these findings, over-representations of sequence polymorphisms in defense-related genes or genes involved in signaling were previously identified in different accessions of *A. thaliana* [17], [100]. Also the over-representation of deletions or sequence deviations in genes involved in ATP-binding, such as genes encoding for transporters or enzymes, might represent a functional adaptation to specific environmental conditions [113]. Copy number variations in N-actetylglucosamine deacetylases were recently reported for *A. thaliana* grown under different temperature conditions [114].

In summary, the five identified gene clusters with increased rate of deletions or sequence deviations indicate a rapid evolutionary change between C24 and Col-0. All genes affected by deletions or sequence deviations are provided in Table S2, and genes affected by amplifications are provided in Table S3.

#### Superfamily analysis of transposons

**Figure 8:**
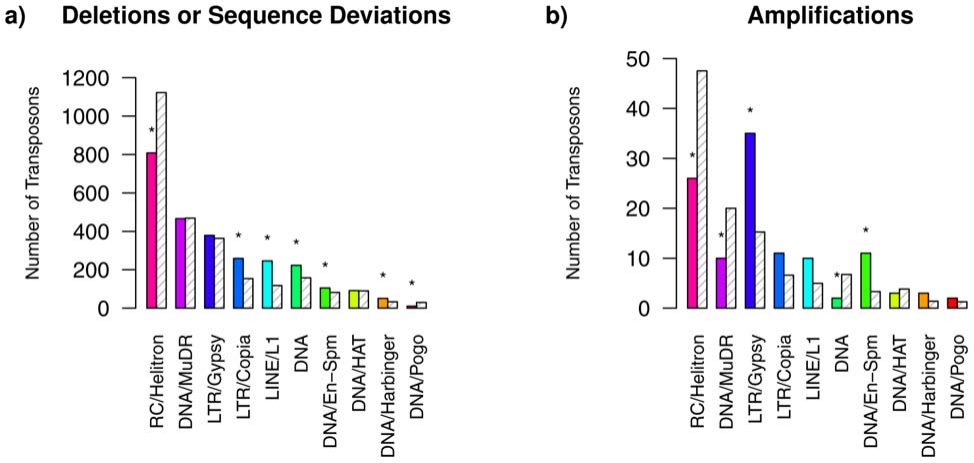
Superfamily classification of transposons in the Arabidopsis Array-CGH data set. Superfamily classification of the 2,695 transposons identified to be affected by deletions or sequence deviations (**a)**) and of the 114 transposons identified to be affected by amplifications (**b)**) under consideration of the TAIR8 transposon annotation. Colored bars show the numbers of affected transposons in each superfamily identified using the state-posterior decoding of the parsimonious fourth-order HMM with underlying state-context tree structure in Figure 5. Grey dashed bars represent the mean number of transposons assigned to these superfamilies for sampling 500 times 2,695 transposons (or 114 transposons) from the total number of transposons of the TAIR8 annotation. Superfamilies highlighted by an asterisk ‘*’ are significantly different (over- or under-represented) with p-values less than 0.01 in comparison to random sampling.

A superfamily classification of transposons affected by deletions or sequence deviations and of transposons affected by amplifications was performed using the TAIR8 transposon annotation of Col-0 to identify under- or over-representations of specific transposon superfamilies in C24. This analysis was done in comparison to randomly sampled control sets of transposons. The results are summarized in Figure 8.

Retrotransposons (LTR/Copia, LINE/L1) moving by a RNA-mediated copy-and-paste mechanism and DNA transposons (DNA, DNA/En-Spm,DNA/Harbinger) moving by a DNA-mediated cut-and-paste mechanism are significantly over-represented among the 2,695 transposons identified as being affected by deletions or sequence deviations in C24 with respect to the reference genome of Col-0. DNA transposons (RC/Helitron, DNA/Pogo) are significantly under-represented among the 2,695 affected transposons.

For transposons affected by amplifications, retrotransposons (LTR/Gypsy) and DNA transposons (DNA/En-Spm) are significantly over-represented among the 114 transposons identified as being affected by amplifications in C24. DNA transposons (RC/Helitron, DNA/MuDR, DNA) are significantly under-represented among these 114 transposons.

Thus, these results indicate that some transposon superfamilies tend to play a more prevalent role for driving the evolution of genomic differences between C24 and Col-0. All these transposons represent fundamental components of *A. thaliana* genomes contributing to size, structure, and variation of genomes [115], [116]. Table S4 provides all transposons identified to be affected by deletions or sequence deviations, and transposons affected by amplifications are contained in Table S5.

### Conclusions

The development of parsimonious higher-order HMMs for the analysis of Array-CGH data has been motivated by the observation of strong spatial dependencies between measurements in close chromosomal proximity. A parsimonious higher-order HMM represents an interpolation between a mixture model ignoring spatial dependencies and a higher-order HMM exhaustively modeling spatial dependencies. To enable this interpolation, the mathematical theory of widely used first-order HMMs has been extended. A central point is the extension of the Bayesian Baum-Welch training by incorporating a dynamic programming approach [98], [99] enabling a data-dependent modeling of spatial dependencies.

In a detailed study based on Array-CGH data for comparing the genomes of the *Arabidopsis thaliana* accessions C24 and Col-0, parsimonious higher-order HMMs clearly improved the identification of deletions or sequence deviations in comparison to typically used first-order HMMs and other existing methods. Especially, parsimonious HMMs of order three up to five with clearly reduced model complexities in comparison to corresponding higher-order HMMs reached the best results.

In-depth functional analyses of identified DNA polymorphisms revealed that most of these genomic differences between C24 and Col-0 are caused by transposons. Genic regions as well as 5′ and 3′ untranslated regions are less affected, but still genes with functions in ATP-binding, cellular signaling, or cell pathogen defense have been found to be specifically affected by deletions or sequence deviations in C24 in comparison to the reference genome of Col-0. These findings are in accordance with other studies [17], [100] and might indicate specific environmental adaptations of both accessions. Additionally, a superfamily classification of transposons has revealed that specific retrotransposon and DNA transposon superfamilies tend to be more involved than others in driving the evolution of C24 and Col-0.

Additional model evaluations performed on widely considered human cell lines showed that parsimonious HMMs are also well-suited for the analysis of non-plant-specific Array-CGH data sets.

All these results indicate that parsimonious higher-order HMMs are useful tools for the analysis of Array-CGH data. Potential future applications could include other domains in which standard first-order HMMs are frequently used. This might include the HMM -based analysis of ChIP-chip data [117]–[120] or the analysis of next-generation sequencing data [121]–[125].

## Supporting Information

Table S1 Arabidopsis Array-CGH data set including detected DNA polymorphisms identified by the parsimonious fourth-order HMM using the state-posterior decoding algorithm.
(TXT)

Table S2 Genes affected by deletions or sequence deviations in C24.
(TXT)

Table S3 Genes affected by amplifications in C24.
(TXT)

Table S4 Transposons affected by deletions or sequence deviations in C24.
(TXT)

Table S5 Transposons affected by amplifications in C24.
(TXT)

Text S1 Mathematical basics of prior distributions for initial state and emission parameters and details to chosen prior parameters are given in the section ‘Prior distribution’. A detailed derivation of the Bayesian Baum-Welch algorithm for a parsimonious higher-order HMM is given in the section ‘Bayesian Baum-Welch algorithm’. Details to the case study on human cell lines are given in the section ‘Model evaluations on human cell lines’. The supporting Figures S1, S2, S3, S4, S5, S6, S7, S8, S9 are provided in the section ‘Supporting Figures’.
(PDF)
