# Supplementary material for: Parsimonious Higher-Order Hidden Markov Models for Improved Array-CGH Analysis with Applications to Arabidopsis thaliana
Source: PLoS Comput Biol. 2012 Jan 12;8(1):e1002286. doi: 10.1371/journal.pcbi.1002286 (PMC3257270; doi:10.1371/journal.pcbi.1002286)
Supplement: Text S1 — Mathematical basics of prior distributions for initial state and emission parameters and details to chosen prior parameters are given in the section ‘Prior distribution’. A detailed derivation of the Bayesian Baum-Welch algorithm for a parsimonious higher-order HMM is given in the section ‘Bayesian Baum-Welch algorithm’. Details to the case study on human cell lines are given in the section ‘Model evaluations on human cell lines’. The supporting Figures S1, S2, S3, S4, S5, S6, S7, S8, S9 are provided in the section ‘Supporting Figures’. (PDF) [file pcbi.1002286.s006.pdf]

Supporting Information — Text S1

Parsimonious higher-order Hidden Markov Models for improved Array-CGH analysis with applications to *Arabidopsis thaliana*

Michael Seifert, André Gohr, Marc Strickert, and Ivo Grosse

Contact: seifert@ipk-gatersleben.de

Contents

1 Prior distribution **3**

1.1 Prior for initial state distribution 3

1.2 Prior for emission parameters 3

1.3 Choice of prior parameters 4

2 Bayesian Baum-Welch algorithm **5**

2.1 Basics of the Bayesian Baum-Welch algorithm 5

2.2 Baum's auxiliary function 5

2.3 Estimation of initial state probabilities 6

2.4 Estimation of transition probabilities 6

2.4.1 Scoring scheme for tree structures 7

2.4.2 Estimating transition probabilities of a set of equivalent state-contexts . . 8

2.4.3 Extended state-context tree 9

2.4.4 Dynamic programming approach 9

2.4.5 Computational complexity of the dynamic programming approach 10

2.5 Estimation of emission parameters 11

3 Model evaluations on human cell lines **12**

3.1 Figure A: Initial model comparisons on human cell lines 13

3.2 Figure B: Different state-context tree structures on human cell lines 14

3.3 Table S1: Overview of run-times of different methods for the analysis of human cell lines 14

4 Supporting Figures **15**

4.1 Figure S1: Number of different state-context trees 15

4.2 Figure S2: Three-state architecture 16

4.3 Figure S3: Choice of model order 17

4.4 Figure S4: Identification of deletions and sequence deviations in the Arabidopsis Array-CGH data set 18

4.5 Figure S5: False-positive-rates for the identification of deletions and sequence deviations in the Arabidopsis Array-CGH data set by parsimonious HMMs at fixed true-positive-rates 19

4.6 Figure S6: Identification of deletions and sequence deviations in the Arabidopsis Array-CGH data set for a less restrictive mapping of validation data 20
4.7 Figure S7: Comparison of a parsimonious fourth-order HMM to existing methods on the Arabidopsis Array-CGH data set based on a less restrictive mapping of validation data 21
4.8 Figure S8: Comparison of a parsimonious fourth-order HMM against a standard first-order HMM on the Arabidopsis Array-CGH data set 22
4.9 Figure S9: Model evaluation on Array-CGH data of human cell lines by Snijders et al. (2001) 23

1 Prior distribution

A problem-specific characterization of the parameters of a parsimonious higher-order HMM λ is achieved by including prior knowledge about data into the training of the model. This prior knowledge is integrated by defining the prior distribution

$$P[\,\lambda\,|\,\Theta\,] := D_1(\,\vec{\pi}\,|\,\Theta_1\,) \cdot D_2(\,A\,|\,\Theta_2\,) \cdot D_3(\,B\,|\,\Theta_3\,) \tag{1}$$

over the model parameters $\lambda := (\vec{\pi}, A, B)$ in dependency of the corresponding hyper-parameters $\Theta := (\Theta_1, \Theta_2, \Theta_3)$. This prior is defined as a product of three independent priors for the initial state distribution $\vec{\pi}$, the set of transition matrices A, and the emission parameters B. The prior $D_2(\,A\,|\,\Theta_2\,)$ for the set of transition matrices is specified in detail in the section 'Prior Distribution' of the manuscript. Thus, only the prior distributions for the initial state distribution and the emission parameters require further considerations.

1.1 Prior for initial state distribution

The prior distribution for the initial state distribution $\vec{\pi}$ with initial state probability $\pi_i := \exp(\Lambda_{\pi_i})$ is defined by a commonly used transformed Dirichlet distribution

$$D_1(\,\vec{\pi}\,|\,\Theta_1\,) := Z(\Theta_1) \prod_{i \in S} \exp(\Lambda_{\pi_i} \cdot \vartheta_i) \tag{2}$$

with hyper-parameter vector $\Theta_1 := (\vartheta_i)_{i \in S}$ and $\vartheta_i \in \mathbb{R}^+$. The normalization constant is represented by $Z(\Theta_1) := \Gamma(\sum_{i \in S} \vartheta_i) / \prod_{i \in S} \Gamma(\vartheta_i)$ with Gamma function $\Gamma(x) = \int_0^\infty u^{x-1} \cdot \exp(-u)\, du$ for all $x \in \mathbb{R}^+$. This transformed Dirichlet prior has been specified in a general form in [1].

1.2 Prior for emission parameters

As prior for the state-specific emission parameters B a product of independent Gaussian-Inverted-Gamma distributions

$$D_3(\,B\,|\,\Theta_3\,) := \prod_{i \in S} N(\,\mu_i\,|\,\Theta_3\,) \cdot I_G(\,\sigma_i\,|\,\Theta_3\,) \tag{3}$$

is used with respect to the hyper-parameter matrix $\Theta_3 := (\eta_i, \epsilon_i, r_i, \alpha_i)_{i \in S}$ containing the mean $\eta_i \in \mathbb{R}$, the scale parameter $\epsilon_i \in \mathbb{R}^+$, the shape parameter $r_i \in \mathbb{R}^+$, and the scale parameter $\alpha_i \in \mathbb{R}^+$. The Gaussian-Inverted-Gamma distribution [2] for state $i \in S$ is defined by a Gaussian distribution

$$N(\,\mu_i\,|\,\Theta_3\,) := \frac{\sqrt{\epsilon_i}}{\sqrt{2\pi}\sigma_i} \exp\left(-\frac{\epsilon_i}{2}\left(\frac{\mu_i - \eta_i}{\sigma_i}\right)^2\right)$$

with mean η_i and standard deviation $\sigma_i/\sqrt{\epsilon_i}$ as prior distribution for the state-specific mean μ_i, and an Inverted-Gamma distribution

$$I_G(\,\sigma_i\,|\,\Theta_3\,) := \frac{2{\alpha_i}^{r_i}}{\Gamma(r_i){\sigma_i}^{2r_i+1}} \exp\left(-\frac{\alpha_i}{{\sigma_i}^2}\right)$$

with shape parameter r_i and scale parameter α_i as prior of the state-specific standard deviation σ_i.

1.3 Choice of prior parameters

The prior defined in Eqn. (1) provides the basics to integrate prior knowledge about the distribution of Array-CGH measurements into a parsimonious higher-order HMM for distinguishing between genomic regions affected by DNA polymorphisms (deletions or sequence deviations, amplifications) and unchanged regions. A histogram of log-ratios (e.g. Figure 2a in the manuscript) helps to characterize the three states of the model in Figure S2 by appropriate emission parameters. The following hyper-parameters have been considered in the case studies with a parsimonious HMM of order L on the Arabidopsis Array-CGH data.

The mean values of the Gaussian emission densities of the emission prior have been set to the values $\eta_- = -3$, $\eta_= = 0$, and $\eta_+ = 1.5$ according to the state-specific mean values $\mu_- = -3$, $\mu_= = 0$, and $\mu_+ = 1.5$ initially defined for the Gaussian emission densities of the model. The corresponding scale parameters have been set to $\epsilon_- = \epsilon_= = 1$ and $\epsilon_+ = 5,000$ providing more flexibility for the training of the means of the Gaussian emission densities of the states '$-$' and '$=$' than for the state '$+$' with respect to the asymmetric distribution of log-ratios having a long negative tail peaking around zero. The shape parameter $r_i = T/2$ and the scale parameter $\alpha_i = Ts^2/2$ for the standard deviation of the Gaussian emission density of state $i \in S$ have been set in dependence of the number of log-ratios $T = 364,339$ and their standard deviation $s = 0.67$.

All parameters of the initial state prior distribution have been set to $\vartheta_i = 3^L$ for each initial state $i \in S$. For each transition matrix A_{τ_l} with $l \in \{1, \ldots, L\}$, the hyper-parameters of the corresponding transition prior $D_2^l(A_{\tau_l} \,|\, \Theta_2^l)$ (see Eqn. (2) in the manuscript) have been set to $\vartheta_{ij} = 3^{L-l}$ for a transition from each state-context $i \in S^l$ to each next state $j \in S$.

To interpolate with parsimonious higher-order HMMs between a mixture model and higher-order HMMs, the following logarithmic values of the tree structure hyper-parameter φ of the tree structure prior $D_2^l(\tau_l \,|\, \varphi)$ defined in Eqn. (3) of the manuscript have been considered. Completely fused trees (e.g. Figure 3a in the manuscript) underlying a mixture model have been obtained for $\log(\varphi) = -30,000$ at latest. The range of $\log(\varphi)$ from $-23,000$ up to 0 has been considered to obtain parsimonious trees (e.g. Figure 3b or Figure 4 in the manuscript). This range has been sampled in steps of $1,000$ for $-23,000$ to $-1,000$, in steps of 100 for $-1,000$ to -100, and finally from -100 up to 0 in steps of 10. Complete trees underlying a higher-order HMM have been obtained for $\log(\varphi) = 10$ at latest (e.g. Figure 3c in the manuscript).

The choice of prior hyper-parameters is generally depending on the characteristics and the size of the data set. The chosen hyper-parameter values provided a good basis for a broad range of parsimonious higher-order HMMs of different model complexities. Basic settings for the emission prior can easily be made via a histogram of log-ratios. No data-specific prior knowledge on transition parameters has been integrated. Characteristic transition parameters of each state have been learned from the data based on the well-characterized emission distributions of a parsimonious higher-order HMM. Best-performing parsimonious models have been found for values of the tree structure hyper-parameter $\log(\varphi)$ in the interval between -100 and 0 representing parsimonious HMMs with clearly reduced model complexities in comparison

to complete higher-order HMMs.

2 Bayesian Baum-Welch algorithm

2.1 Basics of the Bayesian Baum-Welch algorithm

The Bayesian Baum-Welch algorithm is a training procedure that iteratively determines new model parameters

$$\lambda(h+1) := \underset{\lambda}{\textit{argmax}}\, (Q(\,\lambda\,|\,\lambda(h)\,) + \log(P[\,\lambda\,|\,\Theta\,]))$$

for a parsimonious higher-order HMM based on the parameters of the current model $\lambda(h)$ ($h = 1$ initial model) by maximizing Baum's auxiliary function $Q(\,\lambda\,|\,\lambda(h)\,)$ in combination with the prior distribution $P[\,\lambda\,|\,\Theta\,]$ defined in (1). This Bayesian Baum-Welch algorithm performs a maximum a posterior (MAP) estimate of model parameters instead of a maximum likelihood (ML) estimate obtained by the Baum-Welch algorithm. In the following, Baum's auxiliary function is considered in detail and splitted into individual functions that enable the estimation of initial state, transition, and emission parameters of the parsimonious higher-order HMM $\lambda(h+1)$.

2.2 Baum's auxiliary function

Baum's auxiliary function provides the basis for the estimation of the model parameters using the standard Baum-Welch algorithm. This function is defined by

$$Q(\,\lambda\,|\,\lambda(h)\,) := \sum_{k=1}^{K} \sum_{\vec{q} \in S^{T_k}} P[\,\vec{q}\,|\,\vec{o}(k), \lambda(h)\,] \cdot \log(P[\,\vec{o}(k), \vec{q}\,|\,\lambda\,])$$

in analogy to [3]. Here, the complete-data-likelihood of an emission sequence $\vec{o}(k) = (o_1(k), \ldots, o_{T_k}(k))$ and a corresponding state sequence $\vec{q} = (q_1, \ldots, q_{T_k})$ is given by

$$P[\,\vec{o}(k), \vec{q}\,|\,\lambda\,] := \pi_{q_1} \cdot \prod_{t=1}^{L-1} a_{\xi(q_1,\ldots,q_t)q_{t+1}} \prod_{t=L}^{T_k-1} a_{\xi(q_{t-L+1},\ldots,q_t)q_{t+1}} \cdot \prod_{t=1}^{T_k} b_{q_t}(o_t(k))$$

under a parsimonious HMM of order L with parameters $\lambda := (\vec{\pi}, A, B)$. The initial state distribution $\vec{\pi} := (\pi_i)_{i \in S}$, the set of transition matrices $A := \{A_{\tau_1}, \ldots, A_{\tau_L}\}$, and the emission parameters $B := (\mu_i, \sigma_i)_{i \in S}$ are specified in more detail in the section 'Parsimonious Higher-Order Hidden Markov Models' of the manuscript. In addition to this, the function $\xi(q)$ defines the corresponding set of equivalent state-contexts $\xi \in \tau_l$ to which a state-context $q \in S^l$ is belonging under consideration of the corresponding state-context tree τ_l that is underlying the transition matrix A_{τ_l}. Based on the complete-data-likelihood, Baum's auxiliary function can be split up into three independent functions

$$Q(\,\lambda\,|\,\lambda(h)\,) := Q_1(\,\vec{\pi}\,|\,\lambda(h)\,) + Q_2(\,A\,|\,\lambda(h)\,) + Q_3(\,B\,|\,\lambda(h)\,)$$

for representing each class of model parameters separately. Subsequently, the derivation of these three functions and the corresponding model parameter estimations are considered in detail.

2.3 Estimation of initial state probabilities

To estimate the initial state probabilities for the parsimonious higher-order HMM $\lambda(h+1)$, Baum's auxiliary function for estimating the initial state probabilities is required. This function is given by

$$\begin{aligned} Q_1(\,\vec{\pi}\,|\,\lambda(h)\,) &:= \sum_{k=1}^{K} \sum_{\vec{q}\in S^{T_k}} P[\,\vec{q}\,|\,\vec{o}(k),\lambda(h)\,] \cdot \log(\pi_{q_1}) \\ &= \sum_{i\in S} \log(\pi_i) \sum_{k=1}^{K} P[\,q_1 = i\,|\,\vec{o}(k),\lambda(h)\,] \\ &= \sum_{i\in S} \Lambda_{\pi_i} \sum_{k=1}^{K} \gamma_1^k(i) \end{aligned}$$

based on expressing the sum over all state sequences $\vec{q}\in S^{T_k}$ by two sums. The first sum considers all initial states $i\in S$, and the second sum marginalizes over all state sequences $\vec{q}\in S^{T_k}$ with initial state $q_1 = i$ leading to $P[\,q_1 = i\,|\,\vec{o}(k),\lambda(h)\,]$, which represents the state-posterior $\gamma_1^k(i)$ computed under the current parsimonious higher-order HMM $\lambda(h)$ using extended versions of the Forward and Backward algorithm [4]. Finally, the initial state probability π_i is parameterized in the log-space by $\Lambda_{\pi_i} := \log(\pi_i)$.
The new initial state probability $\pi_i^{(h+1)}$ of state $i\in S$ is determined by combining Baum's auxiliary function for initial state parameters with the corresponding prior distribution $D_1(\,\vec{\pi}\,|\,\Theta_1\,)$ defined in (2) with respect to the constraint $\sum_{i\in S}\exp(\Lambda_{\pi_i}) = 1$. This is done by applying the method of Lagrange multipliers to the auxiliary function $Q_1(\,\vec{\pi}\,|\,\lambda(h)\,) + \log(D_1(\,\vec{\pi}\,|\,\Theta_1\,)) - \delta\cdot((\sum_{i\in S}\exp(\Lambda_{\pi_i})) - 1)$ with variable Λ_{π_i} and Lagrange multiplier δ. This leads to the new initial state probability

$$\pi_i^{(h+1)} = \frac{\left(\sum_{k=1}^{K}\gamma_1^k(i)\right) + \vartheta_i}{\left(\sum_{v\in S}\sum_{k=1}^{K}\gamma_1^k(v)\right) + \left(\sum_{v\in S}\vartheta_v\right)}$$

of state $i\in S$ for the parsimonious higher-order HMM $\lambda(h+1)$.

2.4 Estimation of transition probabilities

For estimating the transition probabilities of the next parsimonious higher-order $\lambda(h+1)$, Baum's auxiliary function for the set of transition matrices is required. This function has been specified by $Q_2(\,A\,|\,\lambda(h)\,) := \sum_{l=1}^{L} Q_2^l(\,A_{\tau_l}\,|\,\lambda(h)\,)$ in the section 'Bayesian Baum-Welch Training' of the manuscript. In addition to this, Baum's auxiliary function $Q_2^l(\,A_{\tau_l}\,|\,\lambda(h)\,)$ for transition parameters of the transition matrix A_{τ_l} has been defined in Eqn. (4) of the manuscript. Here, the

derivation of $Q_2^l(\, A_{\tau_l} \,|\, \lambda(h)\,)$ for state-contexts of length $l = L$ is given. This function is specified by

$$
\begin{aligned}
Q_2^L(\, A \,|\, \lambda(h)\,) &:= \sum_{k=1}^{K} \sum_{t=L}^{T_k-1} \sum_{\vec{q} \in S^{T_k}} P[\, \vec{q} \,|\, \vec{o}(k), \lambda(h)\,] \cdot \log\left(a_{\xi(q_{t-L+1},\dots,q_t)q_{t+1}}\right) \\
&= \sum_{\xi \in \tau_L} \sum_{j \in S} \log\left(a_{\xi j}\right) \sum_{k=1}^{K} \sum_{t=L}^{T_k-1} P[\, \vec{q}_{t-L+1\dots t} \in \xi, q_{t+1} = j \,|\, \vec{o}(k), \lambda(h)\,] \\
&= \sum_{\xi \in \tau_L} \sum_{j \in S} \log(a_{\xi j}) \sum_{k=1}^{K} \sum_{t=L}^{T_k-1} \sum_{i \in \xi} P[\, \vec{q}_{t-L+1\dots t} = i, q_{t+1} = j \,|\, \vec{o}(k), \lambda(h)\,] \\
&= \sum_{\xi \in \tau_L} \sum_{j \in S} \varLambda_{a_{\xi j}} \sum_{k=1}^{K} \sum_{t=L}^{T_k-1} \sum_{i \in \xi} \varepsilon_t^k(i,j)
\end{aligned}
$$

substituting the sum over all state sequences $\vec{q} \in S^{T_k}$ by three sums. Two of these sums are shown explicitly and the third sum is substituted as explained subsequently. The first sum considers each set of equivalent state-contexts $\xi \in \tau_L$. The second sum considers each next state $j \in S$. Now, a third sum is necessary to marginalize over all state sequences $\vec{q} \in S^{T_k}$ with fixed current state-context $\vec{q}_{t-L+1\dots t} := (q_{t-L+1}, \dots, q_t) \in \xi$ and fixed next state $q_{t+1} = j$. The resulting marginal distribution $P[\, \vec{q}_{t-L+1\dots t} \in \xi, q_{t+1} = j \,|\, \vec{o}(k), \lambda(h)\,]$ is split up into its individual probabilities $P[\, \vec{q}_{t-L+1\dots t} = i, q_{t+1} = j \,|\, \vec{o}(k), \lambda(h)\,]$ by summing over all state-contexts $i \in \xi$. This probability is denoted by $\varepsilon_t^k(i,j) := P[\, \vec{q}_{t-L+1\dots t} = i, q_{t+1} = j \,|\, \vec{o}(k), \lambda(h)\,]$ that is computed under the current parsimonious higher-order HMMs $\lambda(h)$ using extended versions of the Forward and Backward algorithm [4]. Finally, the transition probability $a_{\xi j}$ is parameterized in the log-space by $\varLambda_{a_{\xi j}} := log(a_{\xi j})$. Baum's auxiliary function $Q_2^l(\, A_{\tau_l} \,|\, \lambda(h)\,)$ for the transition parameters of the initial time steps $l \in \{1, \dots, L-1\}$ can be obtained in analogy to this derivation. How all these functions are used to determine the underlying optimal state-context trees and corresponding optimal transition parameters is outlined in the following.

2.4.1 Scoring scheme for tree structures

The objective function for the computation of an optimal state-context tree τ_l and its corresponding transition matrix A_{τ_l} is specified by

$$
F(\, A_{\tau_l}\,) := \sum_{\xi \in \tau_l} f_l(\, \vec{a}_\xi\,) \tag{4}
$$

in terms of a scoring function $f_l(\, \vec{a}_\xi\,)$ for evaluating each existing set of equivalent state-contexts ξ based on its corresponding transition probabilities $\vec{a}_\xi := (a_{\xi j})_{j \in S}$. This scoring function combines Baum's auxiliary function of transition parameters $Q_2^l(\, A_{\tau_l} \,|\, \lambda(h)\,)$ with the corresponding transition prior $D_2^l(\, A_{\tau_l} \,|\, \Theta_2^l\,)$ and the tree structure prior $D_2^l(\, \tau_l \,|\, \varphi\,)$ defined in the Eqns. (4), (2), and (3) of the manuscript. By regrouping and conflating of individual terms in $Q_2^l(\, A_{\tau_l} \,|\, \lambda(h)\,) + \log(D_2^l(\, A_{\tau_l} \,|\, \Theta_2^l\,)) + \log(D_2^l(\, \tau_l \,|\, \varphi\,))$, the scoring function

$$
f_l(\, \vec{a}_\xi\,) := h_l(\, \vec{a}_\xi\,) + \log(\varphi) + \log(Z(\Theta_{2,\xi}^l)) \tag{5}
$$

for a set of equivalent state-contexts ξ is obtained. This function consists of a function $h_l(\vec{a}_\xi)$, the constant value $\log(\varphi)$ of the tree structure prior, and the corresponding normalization constant $\log(Z(\Theta_{2,\xi}^l))$ of the transition prior. The scoring function is used to determine the score of any existing set of equivalent state-contexts of length l. The score of each equivalence class is maximized by estimating the corresponding optimal transition probabilities for $f_l(\vec{a}_\xi)$. For doing this, its sufficient to consider the function

$$h_l(\vec{a}_\xi) := \begin{cases} \sum_{j \in S} \varLambda_{a_{\xi j}} \left(\left(\sum_{k=1}^{K} \sum_{i \in \xi} \varepsilon_l^k(i,j) \right) + \vartheta_{\xi j} \right) & 1 \leq l < L \\ \sum_{j \in S} \varLambda_{a_{\xi j}} \left(\left(\sum_{k=1}^{K} \sum_{t=L}^{T_k - 1} \sum_{i \in \xi} \varepsilon_t^k(i,j) \right) + \vartheta_{\xi j} \right) & l = L \end{cases} \quad (6)$$

representing the log-transition probability $\varLambda_{a_{\xi j}} := \log(a_{\xi j})$ that needs to be estimated for maximizing $f_l(\vec{a}_\xi)$. As described in the previous section, the probability $\varepsilon_t^k(i,j)$ is computed under the current parsimonious higher-order HMM $\lambda(h)$, and $\vartheta_{\xi j}$ represents the corresponding hyperparameter defined for the transition prior.

Subsequently, the estimation of the transition probabilities for a set of equivalent state-contexts is considered. Based on this, a high-level view of the dynamic programming approach in [5, 6] for maximizing $F(A_{\mathcal{T}_l})$ in (4) is given to provide an overview how an optimal state-context tree and corresponding transition probabilities are computed efficiently.

2.4.2 Estimating transition probabilities of a set of equivalent state-contexts

The basis for the estimation of the transition probabilities belonging to a set of equivalent state-contexts ξ is given by $h_l(\vec{a}_\xi)$ defined in (6) in dependency of the state-context length l. This function is maximized using the method of Lagrange multipliers in subject to the constraint $\sum_{j \in S} \exp(\varLambda_{a_{\xi j}}) = 1$ specifying that all transition probabilities of the set of equivalent state-contexts have to sum up to one. For a set of equivalent state-contexts of a fixed length $1 \leq l < L$, the optimal transition probability is given by

$$a_{\xi j}^{(*)} = \frac{\left(\sum_{k=1}^{K} \sum_{i \in \xi} \varepsilon_l^k(i,j) \right) + \vartheta_{\xi j}}{\left(\sum_{i \in \xi} \sum_{j \in S} \sum_{k=1}^{K} \varepsilon_l^k(i,j) \right) + \left(\sum_{j \in S} \vartheta_{\xi j} \right)} \quad (7)$$

for a transition from a state-context $i \in \xi$ to a next state $j \in S$ at the fixed time step l. In analogy, a set of equivalent state-contexts of length L has the corresponding optimal transition

probability

$$a_{\xi j}^{(*)} = \frac{\left(\sum_{k=1}^{K} \sum_{t=L}^{T_k - 1} \sum_{i \in \xi} \varepsilon_t^k(i,j) \right) + \vartheta_{\xi j}}{\left(\sum_{i \in \xi} \sum_{j \in S} \sum_{k=1}^{K} \sum_{t=L}^{T_k - 1} \varepsilon_t^k(i,j) \right) + \left(\sum_{j \in S} \vartheta_{\xi j} \right)} \tag{8}$$

for a transition from a state-context $i \in \xi$ to a next state $j \in S$ at time steps $t \geq L$. The obtained transition probabilities maximize $f_l(\vec{a}_\xi)$ in (5) and $h_l(\vec{a}_\xi)$ in (6). This can be proven like outlined in [7]. Subsequently, an extended state-context tree data structure is specified to enable the computation of an optimal state-context tree and its corresponding optimal transition probabilities for the next parsimonious higher-order HMM $\lambda(h+1)$.

2.4.3 Extended state-context tree

To efficiently compute an optimal state-context tree τ_l, a data structure representing all existing sets of equivalent state-contexts of length l is required [5, 6]. This is realized by the extended state-context tree ψ_l of height l defined to have the following properties.

- The root node r of ψ_l in depth 0 is labeled by the set $\mathcal{L}[n] := \{\epsilon\}$ representing the empty word ϵ. Each node v in depth $d_v \in \{1, \ldots, l\}$ is labeled by a non-empty subset $\mathcal{L}[v] \subseteq S$ and is linked to its parent node $\mathcal{V}[v]$ in depth $d_v - 1$. Each node v in depth $d_v \in \{0, \ldots, l-1\}$ has $2^N - 1$ child nodes whose labels represent all non-empty elements of the power set of S. All leaf nodes of ψ_l are in depth l.
- Each leaf v represents a set of equivalent state-contexts $\xi(v) := \{(i_1, i_2 \ldots, i_l) : i_1 \in \mathcal{L}[v], i_2 \in \mathcal{L}[\mathcal{V}[v]], \ldots, \epsilon \in \mathcal{L}[r]\}$ of length l. The state-contexts of leaf v define all combinations of states that are obtained by traversing the path from the leaf node v to the root node r.

The fact that the child nodes of each non-leaf node represent all different non-empty subsets of the power set of S ensures that all different sets of equivalent state-contexts of length l are represented by the extended state-context tree ψ_l. The difference between the extended state-context tree ψ_l and the state-context tree τ_l is that ψ_l contains all different sets of equivalent state-contexts, while τ_l represents all state-contexts of length l by a specific set of disjoint sets of state-contexts.

Subsequently, the extended state-context tree ψ_l is transformed into an optimal state-context tree τ_l by selecting the optimal set of disjoint sets of state-contexts representing the optimal transition probabilities of the transition matrix A_{τ_l} of the next parsimonious higher-order HMM $\lambda(h+1)$.

2.4.4 Dynamic programming approach

The general scheme of the dynamic programming approach for computing optimal transition parameters has been proposed for parsimonious higher-order Markov models in [5]. Further

refinements for a maximum a posterior estimation providing an optimal state-context tree, its corresponding transition parameters, and more details on an efficient implementation have been described in [6].
For determining the optimal transition parameters of the transition matrix A_{τ_l} of the next parsimonious higher-order HMM $\lambda(h+1)$, this dynamic programming approach is used to maximize the scoring function $F(\,A_{\tau_l}\,)$ in (4). An overview of the computational scheme of this algorithm is given by the following steps.

- *Initialization*: For each leaf node v of the extended state-context tree ψ_l in depth l the corresponding equivalence class ξ of v is considered.
  1. Estimate the optimal transition probabilities $a_{\xi j}^{(*)}$ for each next state $j \in S$ using (7) or (8). Store these probabilities in leaf node v.
  2. Compute the score of the set equivalent state-contexts $f_l(\,\vec{a}_{\xi}^{(*)}\,)$ defined in (5) using the corresponding optimal transition probabilities. Store this score in leaf node v.

- *Iteration*: Climb up one level towards the root. Consider each node v of the extended state-context tree ψ_l in the current depth.
  1. Determine all child nodes of the current node v and consider each combination of child nodes whose labels specify a partition in the set of partitions of S.
  2. Compute the score for each partition by adding the scores stored in the corresponding child nodes. Determine the partition with the maximal score and store this score in the current node v. Delete all sub-trees under v that have a root node which is not required for the partition with the maximal score.
  3. Stop if the current node v is the root node of the extended state-context tree ψ_l, otherwise continue with the next iteration step.

The dynamic programming algorithm iterates bottom-up from the leaf nodes to the root node of the extended state-context tree ψ_l. The initialization step provides the basics for each set of equivalent state-contexts represented by the extended state-context tree. The iteration step transforms the extended state-context tree ψ_l into a state-context tree τ_l by successively determining the optimal partition of states under each non-leaf node of ψ_l. After the iteration step, only the optimal set of disjoint sets of state-contexts remains and the extended state-context tree ψ_l has been transformed into the optimal state-context tree τ_l representing the corresponding optimal transition probabilities of the transition matrix A_{τ_l} of the parsimonious higher-order HMM $\lambda(h+1)$. Details for an efficient recursive implementation are described in [6] and implemented in Jstacs (www.jstacs.de).

2.4.5 Computational complexity of the dynamic programming approach

The computational complexity of the dynamic programming algorithm is derived for the extended state-context tree ψ_L of depth L. Based on this extended tree, the computation of the optimal tree $\tau_L^{(h+1)}$ and its corresponding optimal transition probabilities $a_{\xi j}^{(h+1)}$ for the next parsimonious higher-order HMM $\lambda(h+1)$ has the greatest computational complexity. This is because state-contexts of the fixed maximal length L have to be considered. For the following analysis, the parsimonious HMM of order L is assumed to have N states and the processed

emission sequence is assumed to have a length of T.

In the initialization step, each leaf node of the extended state-context tree ψ_L is evaluated by computing the optimal transition probabilities $a^{(*)}_{\xi j}$ and the optimal score $f_L(\,\vec{a}^{(*)}_{\xi}\,)$ for the equivalence class ξ of each leaf node. Since each non-leaf node of the extended tree ψ_L has exactly $2^N - 1$ child nodes, the initialization step has to operate on $(2^N - 1)^L$ leaf nodes. For each leaf node, the computation of the transition probability $a^{(*)}_{\xi j}$ in (8) for the equivalence class ξ of a leaf node involves at most N^L different probabilities $\varepsilon_t(i, j)$ for each time step t of the T time steps. For each of the $(2^N - 1)^L$ equivalence classes represented by the leaf nodes of the extended tree, N different transition probabilities and the corresponding optimal score have to be computed. This leads to a computational complexity of $O\left((2^N - 1)^L \cdot N^{L+1} \cdot T\right)$ for the initialization step.

The iteration step is working on each non-leaf node of the extended tree ψ_L. The total number of non-leaf nodes of this tree is $((2^N - 1)^L - 1)/((2^N - 1) - 1)$. This follows from the geometric series that develops by the common ratio of $2^N - 1$ child nodes per non-leaf node. For each of these non-leaf nodes all different partitions of the set of hidden states S must be considered to compute the optimal partition and their corresponding optimal score. The number of different partitions is given by the Bell number B_N defining the existing numbers of partions of the N states. The computation of the score of each partition requires at most a sum over N scores stored in the child nodes of a non-leaf node. In addition to this, at most $2^N - 2$ sub-trees of child nodes that are not part of the optimal partition must be removed from each non-leaf node. This leads to a run-time of $O\left(((2^N - 1)^L - 1)/((2^N - 1) - 1) \cdot (B_N \cdot N + 2^N - 2)\right)$ that is mainly influenced by B_N which grows faster than 2^N for $N > 4$.

In summary, the upper bound of the computational complexity of the dynamic programming approach is given by the sum of the computational complexities of the initialization and iteration step.

2.5 Estimation of emission parameters

To estimate the transition parameters for the next parsimonious higher-order HMM $\lambda(h + 1)$, Baum's auxiliary function for the emission parameters is required. This function is defined by

$$
\begin{aligned}
Q_3(\,B \,|\, \lambda(h)\,) :=& \sum_{k=1}^{K} \sum_{t=1}^{T_k} \sum_{\vec{q} \in S^{T_k}} P[\,\vec{q} \,|\, \vec{o}(k), \lambda(h)\,] \cdot \log\left(b_{q_t}(o_t(k))\right) \\
=& \sum_{i \in S} \sum_{k=1}^{K} \sum_{t=1}^{T_k} \log\left(b_i(o_t(k))\right) \cdot P[\,q_t = i \,|\, \vec{o}(k), \lambda(h)\,] \\
=& \sum_{i \in S} \sum_{k=1}^{K} \sum_{t=1}^{T_k} \log\left(b_i(o_t(k))\right) \cdot \gamma^k_t(i)
\end{aligned}
$$

including the substitution of the sum over all state sequences $\vec{q} \in S^{T_k}$ by two sums. The first sum runs over all current states $i \in S$. Now, a second sum is required to marginalize over all state sequences $\vec{q} \in S^{T_k}$ with fixed current state $q_t = i$. The second sum can be simplified to its marginal probability $\gamma^k_t(i) := P[\,q_t = i \,|\, \vec{o}(k), \lambda_L(h)\,]$ representing the state-posterior computed under the current parsimonious higher-order HMM $\lambda(h)$ using extended versions of the Forward

and Backward algorithm [4].
The state-specific mean $\mu_i^{(h+1)}$ and the state-specific standard deviation $\sigma_i^{(h+1)}$ of the Gaussian emission density of state $i \in S$ are determined by maximizing Baum's auxiliary function for emission parameters $Q_3(\, B \,|\, \lambda(h)\,)$ in combination with the emission prior $D_3(\, B \,|\, \Theta_3\,)$ defined in (3). This is done by determining the critical points of the derivatives of $Q_3(\, B \,|\, \lambda(h)\,) + \log(D_3(\, B \,|\, \Theta_3\,))$ with respect to the mean μ_i and the standard deviation σ_i. This leads to the mean

$$\mu_i^{(h+1)} = \frac{\left(\sum_{k=1}^{K}\sum_{t=1}^{T_k} o_t(k) \cdot \gamma_t^k(i)\right) + \epsilon_i \eta_i}{\left(\sum_{k=1}^{K}\sum_{t=1}^{T_k} \gamma_t^k(i)\right) + \epsilon_i}$$

and the standard deviation

$$\sigma_i^{(h+1)} = \sqrt{\frac{\left(\sum_{k=1}^{K}\sum_{t=1}^{T_k} \gamma_t^k(i)\left(o_t(k) - \mu_i^{(h+1)}\right)^2\right) + \epsilon_i(\mu_i^{(h+1)} - \eta_i)^2 + 2\alpha_i}{\left(\sum_{k=1}^{K}\sum_{t=1}^{T_k} \gamma_t^k(i)\right) + 2r_i + 2}}$$

for the Gaussian emission density of each state $i \in S$ of the parsimonious higher-order HMM $\lambda(h+1)$.

3 Model evaluations on human cell lines

Array-CGH data of human cell lines by Snijders et al. [8] frequently considered in other model comparison studies (e.g. [9–13]) were analyzed to evaluate the identification of known monosomies and trisomies by different methods. The Array-CGH profiles of 10 human cell lines (9 fibroblast lines, 1 B-lymphocyte line) and corresponding known underlying monosomies and trisomies of chromosomal regions were taken from the R-package of RJaCGH [12]. In more detail, the cell lines gm01524, gm01750, gm02948, gm03134, gm03563, gm03576, gm04435, gm07081, gm13031, and gm13330 have been included into the analysis. The whole data set comprised 20,885 measurements across the 23 chromosomes of all 10 cell lines. These are about 17.5 times less measurements than contained in the more complex *A. thaliana* data set measured on a high-density tiling array.
We trained parsimonious HMMs on this data set interpolating between a mixture model and a first-order HMM. The transition to parsimonious higher-order HMMs was not necessary, because the first-order HMMs already reached a nearly perfect identification of known monosomies and trisomies. The resulting ROC curves are shown in Figure A. The standard first-order HMM and the parsimonious HMM clearly outperformed the mixture model that does not model any chromosomal dependencies. Since the parsimonious HMM showed to be slightly better than the first-order HMM (PHMM: 100% TPR at 0.167% FPR; HMM: 100% TPR at 0.172% FPR) and since this model has less transition parameters, we further applied this

model for comparisons to other existing methods. Detailed results of this comparison study are reported in the main manuscript and are shown in Figure S9. The parsimonious HMM, but also both Bayesian HMMs, RJaCGH and GHMM (RJaCGH: 100% TPR at 0.275% FPR; GHMM: 100% TPR at 0.196% FPR), reach the best, nearly perfect identification of known chromosomal aberrations in the individual human cell lines. The run-times of the different methods are given in Table S1 of this section.

The analysis of the Snijders data [8] was performed using the data-dependent standard initialization as applied for the Arabidopsis study. We just modified the initial means of the Gaussian emission densities of state '$-$' and '$+$' to -0.5 and 0.5 respectively to account for the different distribution of log-ratios compared to the Arabidopsis data set. We interpolated between the mixture model and the standard first-order HMM by choosing the tree structure hyper-parameter $\log(\varphi) \in \{-10000, -1000, 0\}$. Each parsimonious HMM converged to the identical state-context tree shown in Figure Bb. This tree shows that the state '$-$' modeling monosomies and the state '$+$' modeling trisomies are sharing their transition parameters, while unchanged regions modeled by state '$=$' have their own set of transition parameters. That nicely reflects the occurrence of monosomies and trisomies in the Snijders data. These chromosomal aberrations occur much less frequently than unchanged chromosomal regions (501 monosomies or trisomies compared to 20,384 unchanged regions; 2.46% polymorphic regions).

3.1 Figure A: Initial model comparisons on human cell lines

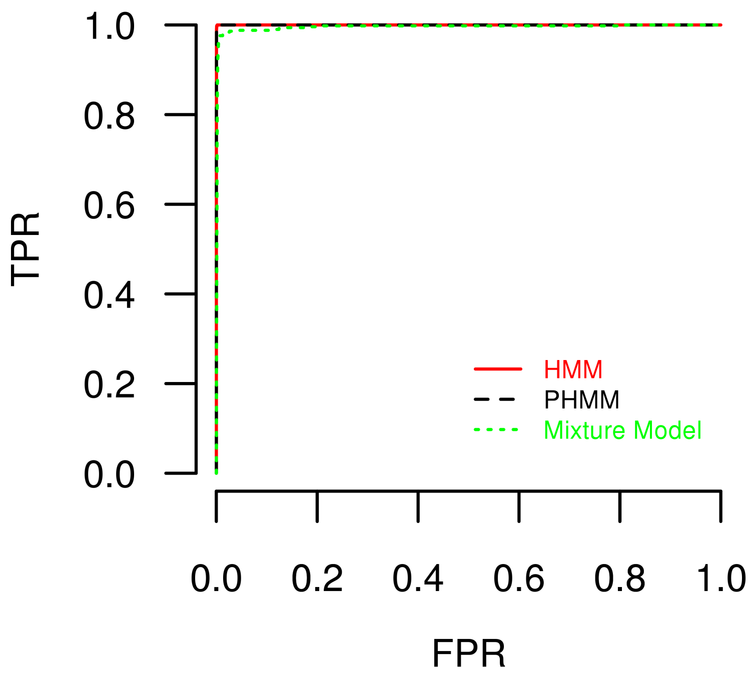

Figure A: ROC curves comparing the identification of known monosomies and trisomies of chromosomal regions in the Snijders data [8]. The standard first-order HMM (red) and the parsimonious first-order HMM (black) outperformed the mixture model (HMM of order zero; green). The parsimonious first-order HMM was also slightly better than the standard first-order HMM by reaching 100% TPR at 0.167% FPR compared to 0.172% FPR for the HMM. The

mixture model reached this performance at 79.9% FPR. The parsimonious first-order HMM has been further considered for the comparison against the other existing methods summarized in Figure S9 of the main manuscript. The different state-context trees underlying the three models are shown in Figure B.

3.2 Figure B: Different state-context tree structures on human cell lines

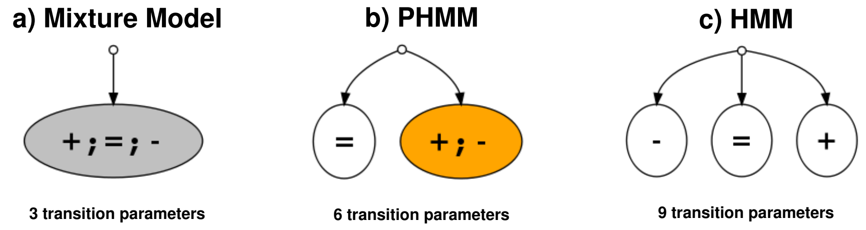

Figure B: Different state-context trees obtained for interpolating between a mixture model (HMM of order zero) and a standard first-order HMM on the Snijders data [8]. Detailed descriptions of state-context trees are given in Figure 3 and Figure 5 of the main manuscript. **a)** The mixture model has the most parsimonious tree representing all state-contexts '$-$', '$=$', and '$+$' by just one leaf node resulting in three different transition parameters that are shared across all three hidden states. **b)** The parsimonious first-order HMM separates all state-contexts '$-$', '$=$', and '$+$ into two different leaf nodes, one for '$=$' and another for '$-$' and '$+$' together. This leads to six different transition parameters, three for state '$=$' and three parameters shared across the states '$-$' and '$+$'. **c)** The standard first-order HMM represents each state-context '$-$', '$=$', and '$+$' in a separate leaf node leading to nine different transition parameters, three for each state.

3.3 Table S1: Overview of run-times of different methods for the analysis of human cell lines

Shortcut	Method	Reference	Computing time
GHMM	Bayesian first-order HMM	[13]	1 min
PHMM	Parsimonious first-order HMM	see main text	1 min
wuHMM	First-order HMM	[14]	1 min
ACE	Analysis of Copy Errors	[15]	2 min
CGHseg	CGH segmentation	[16]	4 min
GLAD	Gain and Loss Analysis of DNA	[11]	4 min
wavelet	Haar wavelet and clustering	[17]	4 min
FHMM	First-order HMM	[9]	6 min
CBS	Circular Binary Segmentation	[10]	8 min
RJaCGH	Bayesian first-order HMM	[12]	70 min

Run-times in minutes required for the analysis of the 10 human cell lines from [8] by the ten different methods. All methods except GHMM, PHMM, wuHMM, and RJaCGH were run on the ADaCGH web-server [18] (AMD Opteron 2.2 GHz CPU with 6 GB RAM). The other methods GHMM, PHMM, wuHMM, and RJaCGH were run on a standard desktop computer with Intel CPU T9500 2.6 GHz and 4 GB RAM.

4 Supporting Figures

4.1 Figure S1: Number of different state-context trees

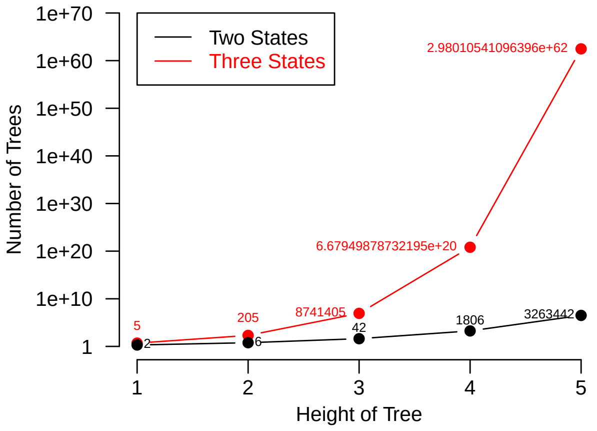

Figure S1: Number of different state-context trees. Increase of the number of different state-context trees for a fixed number of states (black: two; red: three) in dependency of the height of the tree. The numbers of existing trees are plotted in logarithmic scale. Exact numbers are given for all heights of two states and for three states up to height three. Parsimonious higher-order HMMs with three states up to order five have been investigated in the manuscript.

4.2 Figure S2: Three-state architecture

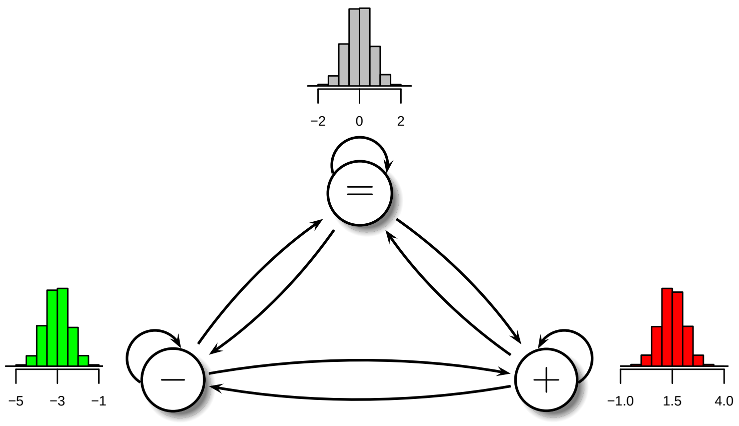

Figure S2: Three-state architecture. The three-state architecture of the parsimonious higher-order HMM used in the manuscript. The states of the model are represented by labeled circles with corresponding state-specific Gaussian emission densities. State '$-$' models chromosomal regions affected by deletions or sequence deviations, state '$=$' models unchanged chromosomal regions, and state '$+$' models chromosomal regions affected by amplifications. Arrows represent possible transitions between states. The corresponding transition probabilities of each transition matrix are represented by state-context trees like illustrated in Figure 3 or Figure 4 of the manuscript.

4.3 Figure S3: Choice of model order

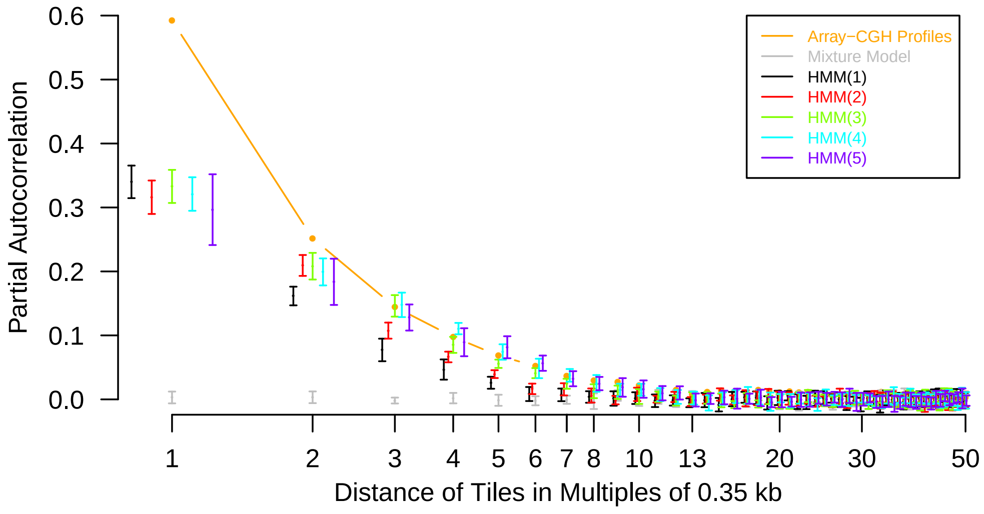

Figure S3: Choice of model order. Modeling of the mean partial autocorrelation function of the Arabidopsis Array-CGH data set (orange) by different models (other colors). The mean partial autocorrelation is computed by adding the partial autocorrelation functions of the five chromosome-specific Array-CGH profiles weighted by their proportion of measurements in relation to the total number of measurements in the Array-CGH data set. Each model was trained with the Arabidopsis Array-CGH data using the Bayesian Baum-Welch algorithm. Then 100 artificial chromosomes with 10,000 log-ratios were sampled from each trained model to compute its mean partial autocorrelations. To ease the comparison, the x-axis is plotted in logarithmic scale and the values of the mean partial autocorrelation function are plotted slightly shifted for each position. As expected from theory and as indicated by mean partial autocorrelations of about zero, the mixture model of three Gaussian densities (grey) does not model dependencies between adjacent chromosomal regions. The first-order HMM (black) represents a natural extension of the mixture model enabling the modeling of dependencies between directly adjacent chromosomal regions indicated by positive values of the mean partial autocorrelation function. Higher-order HMMs of order two up to five (red to purple) clearly improve the modeling of dependencies between adjacent chromosomal regions present in the Arabidopsis Array-CGH data. The trend that HMMs with a higher model order are better able to model the partial autocorrelation function is expected from theory because of their more complex state-transition processes enabling an improved modeling of spatial dependencies compared to HMMs with a smaller model order. However, especially for position one (directly adjacent tiles on a chromosome) all models clearly underestimate the partial autocorrelations of the Array-CGH data set. One reason for this is the difference between the hybridization of DNA segments leading to the log-ratios of the Arabidopsis Array-CGH data set and the sampling of log-ratios from state-specific Gaussian emission densities. The hybridized DNA segments have lengths up to 900 bp. Thus, log-ratios measured for directly adjacent chromosomal regions in distance of about 350 bp are expected to be more similar to each other than log-ratios sampled from a state-specific Gaussian emission density that has to cover a broader range of log-ratios. Although, none of these HMMs was able to perfectly model the partial autocorrelation structure of the Arabidopsis Array-CGH data set. But still, HMMs are flexible models well-suited for the analysis of real Array-CGH profiles.

4.4 Figure S4: Identification of deletions and sequence deviations in the Arabidopsis Array-CGH data set

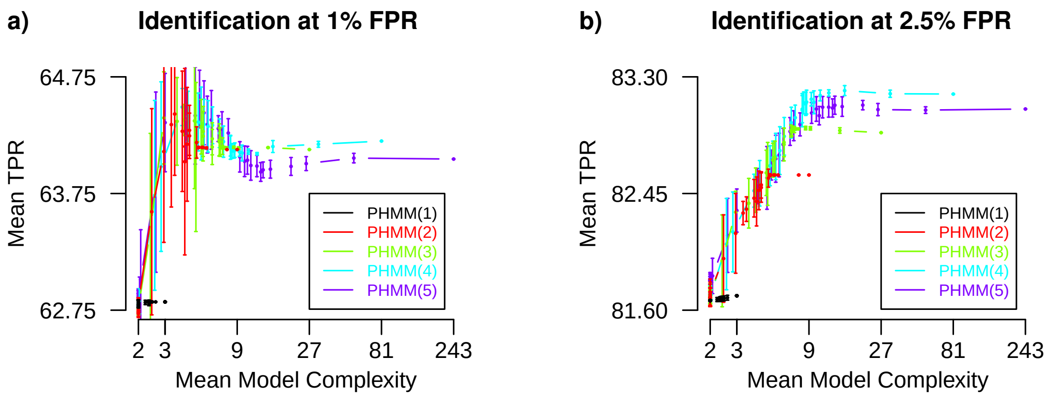

Figure S4: Identification of deletions and sequence deviations in the Arabidopsis Array-CGH data set. This figure shows the content of Figure 4 in the manuscript extended by standard deviations for the obtained mean true-positive-rates (TPRs). The curves show the mean TPRs and corresponding standard deviations for the identification of candidate regions of deletions or sequence deviations at a fixed false-positive-rate (FPR) of 1% (**a)**) and 2.5% (**b)**) obtained by parsimonious HMMs of order $L \in \{1, \ldots, 5\}$ of different model complexities across twenty different initializations. The rightmost point of each curve of parsimonious HMMs of order L (PHMM(L)) represents the corresponding higher-order HMM of order L with highest model complexity of 3^L leaf nodes in the state-context tree underlying the transition matrix A_{τ_L}. The rightmost point of the black curve represents the standard first-order HMM. At both levels of FPRs, parsimonious higher-order HMMs are significantly better than parsimonious HMMs of order one including the standard first-order HMM. At the level of 1%FPR, parsimonious higher-order HMMs with a mean model complexity in the range of 3 up to 9 also identify deletions or sequence deviations better than higher-order HMMs. At 2.5% FPR, clearly reduced model complexities are sufficient to reach identifications of deletions or sequence deviations by parsimonious higher-order HMMs comparable or slightly better than corresponding higher-order HMMs.

4.5 Figure S5: False-positive-rates for the identification of deletions and sequence deviations in the Arabidopsis Array-CGH data set by parsimonious HMMs at fixed true-positive-rates

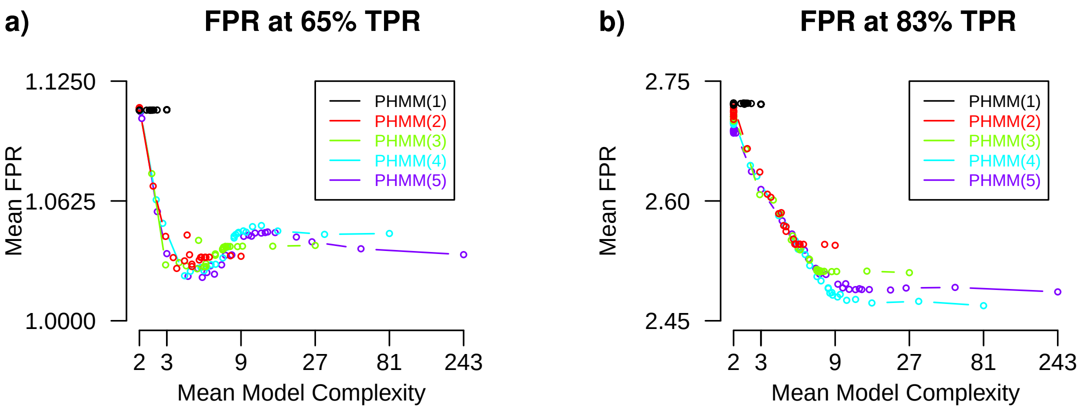

Figure S5: False-positive-rates for the identification of deletions and sequence deviations in the Arabidopsis Array-CGH data set by parsimonious HMMs at fixed true-positive-rates. Curves of mean false-positive-rates (FPRs) for the identification of candidate regions of deletions or sequence deviations at a fixed true-positive-rate (TPR) of 65% (**a)**) and of 83% (**b)**) obtained by parsimonious HMMs of order $L \in \{1, \ldots, 5\}$ of different model complexities across twenty different initializations. The rightmost point of each curve of parsimonious HMMs of order L (PHMM(L)) represents the corresponding higher-order HMM of order L with highest model complexity of 3^L leaf nodes in the state-context tree underlying the transition matrix A_{τ_L}. The rightmost point of the black curve represents the standard first-order HMM. At both levels of TPRs, parsimonious higher-order HMMs are clearly better than parsimonious HMMs of order one including the standard first-order HMM. At the level of 65% TPR, parsimonious higher-order HMMs with a mean model complexity in the range of 3 up to 9 also identify deletions or sequence deviations better than higher-order HMMs. At 83% TPR, clearly reduced model complexities are sufficient to reach identifications of deletions or sequence deviations by parsimonious higher-order HMMs comparable to corresponding higher-order HMMs. TPRs for fixed FPRs in the range of 65% and 83% are shown in the corresponding Figure 4 of the main manuscript.

4.6 Figure S6: Identification of deletions and sequence deviations in the Arabidopsis Array-CGH data set for a less restrictive mapping of validation data

Here, model evaluation is done based on a less restrictive mapping of candidate regions of deletions or sequence deviations identified in the independent array-based resequencing experiment (Clark et al. (2007), Zeller et al. (2008)) described in the manuscript. The candidate regions were used to identify each tile in the Array-CGH data set for which at least 40% of its nucleotides ($\geq$ 24 bp of 60 bp) are covered by candidate regions. This results in 24,231 tiles labeled as being affected by potential deletions or sequence deviations among the 364,339 tiles in the Array-CGH data set. Like shown in Figure 2b for the more restrictive validation data, these labeled tiles also show a clear enrichment of negative log-ratios (histogram not shown). The less restrictive validation data is subsequently considered for model evaluation.

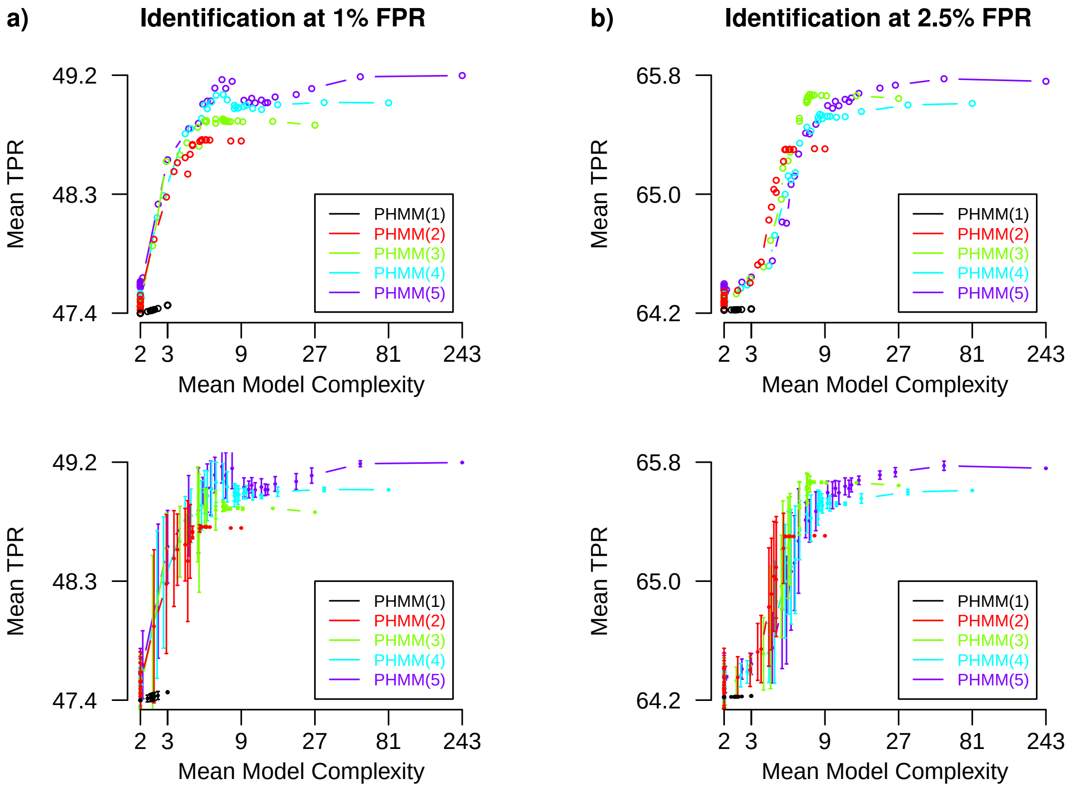

Figure S6: Identification of deletions and sequence deviations in the Arabidopsis Array-CGH data set for a less restrictive mapping of validation data. Curves of mean true-positive-rates (TPRs) for the identification of candidate regions of deletions or sequence deviations at a fixed false-positive-rate (FPR) of 1% (a)) and 2.5% (b)) obtained by parsimonious HMMs of order $L \in \{1, \ldots, 5\}$ of different model complexities across twenty different initializations. The rightmost point of each curve of parsimonious HMMs of order L (PHMM(L)) represents the corresponding higher-order HMM of order L with highest model complexity of 3^L leaf nodes in the state-context tree underlying the transition matrix A_{τ_L}. The rightmost point of the black curve represents the standard first-order HMM. At both levels of FPRs, parsimonious higher-order HMMs are significantly better than parsimonious HMMs of order one including the standard first-order HMM. At the level of 1% FPR, parsimonious higher-order HMMs with a mean model complexity in the range of 3 up to 9 exist that are able to identify deletions or sequence deviations better than higher-order HMMs. At 2.5% FPR, clearly reduced model complexities are sufficient to reach identifications of deletions or sequence deviations by parsimonious higher-order HMMs comparable or slightly better than corresponding higher-order HMMs.

4.7 Figure S7: Comparison of a parsimonious fourth-order HMM to existing methods on the Arabidopsis Array-CGH data set based on a less restrictive mapping of validation data

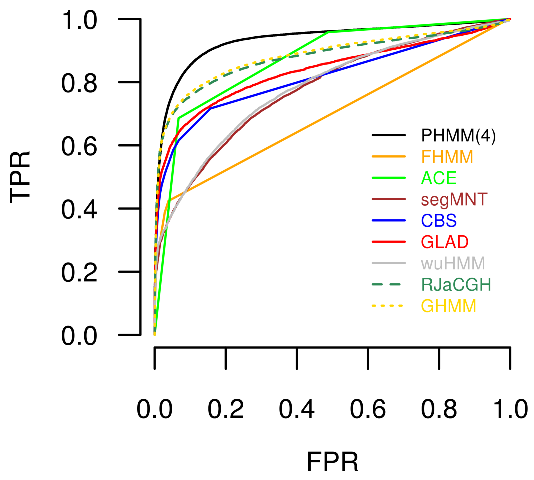

Figure S7: Comparison of a parsimonious fourth-order HMM to existing methods on the Arabidopsis Array-CGH data set based on a less restrictive mapping of validation data. Receiver operating characteristic (ROC) curves of different methods for evaluating the identification of deletions and sequence deviations in the Arabidopsis Array-CGH data set based on the less restrictive validation data described in Figure S6. The methods FHMM, ACE, segMNT, CBS, GLAD, wuHMM, RJaCGH, GHMM, and one of the best performing parsimonious HMMs of order four (see Figure 5 in the manuscript) were used to identify deletions or sequence deviations in the Array-CGH data set. The results for FHMM, ACE, CBS, and GLAD were computed using the ADaCGH webserver with standard settings. For segMNT, wuHMM, RJaCGH, and GHMM, corresponding software packages were used with standard settings. The parsimonious HMM of order four reaches the best identification of deletions or sequence deviations (black). Comparable results on the more restrictive validation data are shown in Figure 6 of the main manuscript.

4.8 Figure S8: Comparison of a parsimonious fourth-order HMM against a standard first-order HMM on the Arabidopsis Array-CGH data set

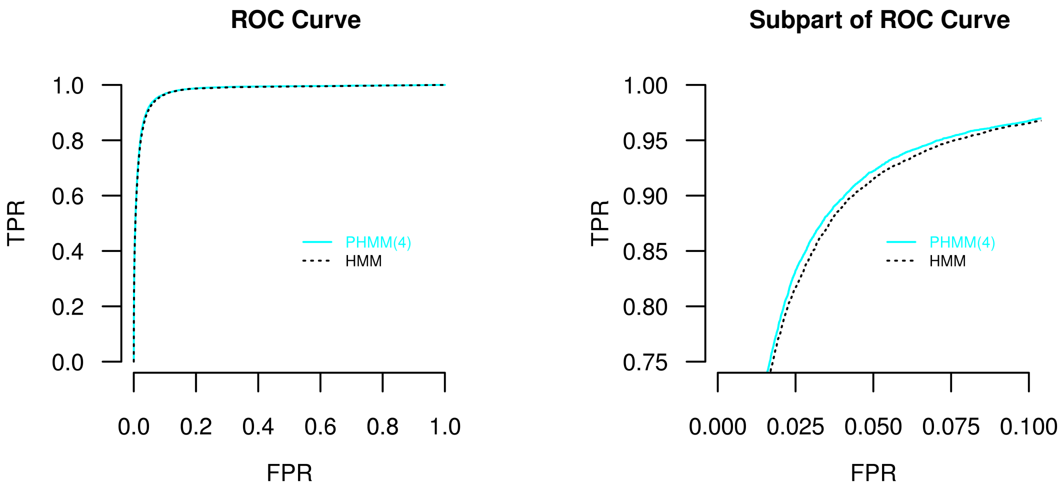

Figure S8: Comparison of a parsimonious fourth-order HMM against a standard first-order HMM on the Arabidopsis Array-CGH data set. Receiver operating characteristic (ROC) curves of the parsimonious fourth-order HMM (cyan) with underlying state-context tree shown in Figure 5 and the standard first-order HMM (black). The left subfigure shows the whole ROC curves and the right subfigure represents a selected subpart of these ROC curves relavant for evaluating the performance of the identifcation of deletions or sequence deviations. The parsimonious fourth-order HMM reaches a better identification than the standard first-order HMM.

4.9 Figure S9: Model evaluation on Array-CGH data of human cell lines by Snijders et al. (2001)

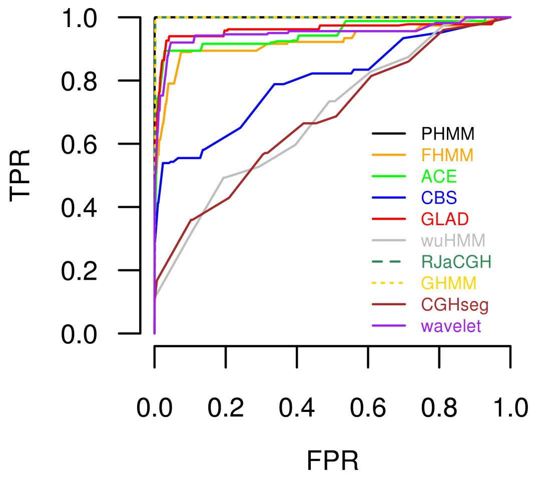

Figure S9: Model evaluation on Array-CGH data of human cell lines by Snijders et al. (2001). Receiver operating characteristic (ROC) curves of different methods for evaluating the identification of known trisomies and monosomies in individual cell lines. The methods FHMM, ACE, CBS, GLAD, wuHMM, RJaCGH, GHMM, CGHseg, wavelet and a parsimonious HMM of order one were used to identify trisomies and monosomies. The results for FHMM, ACE, CBS, GLAD, CGHseg, and wavelet were computed using the ADaCGH webserver with standard settings. For wuHMM, RJaCGH, and GHMM, corresponding software packages were used with standard settings. The two Bayesian HMMs, RJaCGH (green) and GHMM (yellow), and the PHMM (black) reach the best identification of known chromosomal aberrations.

References

[1] MacKay DJC (1998) Choice of Basis for Laplace Approximation. Machine Learning 33: 77-86.

[2] Evans M, Hastings N, Peacock B (2000) Statistical Distributions, 3rd Edition. Wiley Series in Probability and Statistics. John Wiley & Sons, Inc.

[3] Rabiner LR (1989) A Tutorial on Hidden Markov Models and Selected Applications in Speech Recognition. Proc IEEE 77: 257-286.

[4] Seifert M (2010) Extensions of Hidden Markov Modles for the analysis of DNA microarray data. PhD Thesis, University of Halle-Wittenberg, http://nbn-resolving.de/urn:nbn:de:gbv:3:4-4110.

[5] Bourguignon PY, Robelin D (2004) Modèles de Markov parcimonieux. Actes de JOBIM, Montréal, Canada .

[6] Gohr A (2006) The Idea of Parsimony in Tree Based Statistical Models - Parsimonious Markov Models and Parsimonious Bayesian Networks with Applications to Classification of DNA Functional Sites. Diploma Thesis, Martin Luther University Halle-Wittenberg .

[7] Durbin R, Eddy S, Krogh A, Mitchison G (1998) Biological sequence analysis - Probabilistic models of proteins and nucleic acids. Cambridge University Press.

[8] Snijders AM, Nowak N, Segraves R, Blackwood S, Brown N, et al. (2001) Assembly of microarrays for genome-wide measurement of DNA copy number. Nat Genet 29: 263-264.

[9] Fridlyand J, Snijders AM, Pinkel D, Albertson DG, Jain AN (2004) Hidden Markov models approach to the analysis of array CGH data. J Multivariate Anal 90: 132-153.

[10] Olshen AB, Venkatraman ES, Lucito R, Wigler M (2004) Circular binary segmentation for the analysis of array-based DNA copy number data. Biostatistics 5: 557-572.

[11] Hupé P, Stransky N, Thiery JP, Radvanyi F, Barillot E (2004) Analysis of array CGH data: from signal ratio to gain and loss of DNA regions. Bioinformatics 20: 3413-3422.

[12] Rueda OM, Diaz-Uriarte R (2007) Flexible and accurate detection of genomic copy-number changes from aCGH. PLoS Comput Biol 3: e122.

[13] Guha S, Li Y, Neuberg D (2008) Bayesian Hidden Markov Modeling of Array CGH Data. J Amer Statist Assoc 103: 485-497.

[14] Cahan P, Godfrey LE, Eis PS, Richmond TA, Selzer RR, et al. (2008) wuHMM: a robust algorithm to detect DNA copy number variation using oligonucleotide microarray data. Nucleic Acids Res 36: 1-11.

[15] Lingjaerde OC, Baumbusch LO, Liestol K, Glad IG, Borresen-Dale AL (2005) CGH-Explorer: a program for analysis of array-CGH data. Bioinformatics 21: 821-822.

[16] Picard F, Robin S, Lavielle M, Vaisse C, Daudin JJ (2005) A statistical approach for array CGH data analysis. BMC Bioinformatics 6.

[17] Hsu L, Self SG, Grove D, Randolph T, Wang K, et al. (2005) Denoising array-based comparative genomic hybridization data using wavelets. Biostatistics 6: 211-226.

[18] Diaz-Uriarte R, Rueda OM (2007) ADaCGH: A Parallelized Web-Based Application and R Package for the Analysis of aCGH Data. PLoS ONE 2: e737.
